# Synthetic antibodies against BRIL as universal fiducial marks for single−particle cryoEM structure determination of membrane proteins

**DOI:** 10.1038/s41467-020-15363-0

**Published:** 2020-03-27

**Authors:** Somnath Mukherjee, Satchal K. Erramilli, Mark Ammirati, Frances J. D. Alvarez, Kimberly F. Fennell, Michael D. Purdy, Blazej M. Skrobek, Katarzyna Radziwon, John Coukos, Yanyong Kang, Przemysław Dutka, Xiang Gao, Xiayang Qiu, Mark Yeager, H. Eric Xu, Seungil Han, Anthony A. Kossiakoff

**Affiliations:** 1 Department of Biochemistry and Molecular Biology, The University of Chicago, Chicago, IL USA; 2 Medicine Design, Worldwide Research and Development, Pfizer Inc., Eastern Point Road, Groton, CT 06340 USA; 3 Department of Molecular Physiology and Biological Physics, University of Virginia School of Medicine, Charlottesville, VA USA; 4 Center for Cancer and Cell Biology, Structural Biology Program, Van Andel Research Institute, Grand Rapids, MI USA; 5 Key Laboratory of Receptor Research, VARI-SIMM Center, Center for Structure and Function of Drug Targets, Shanghai Institute of Materia Medica, Chinese Academy of Sciences, Shanghai, China; 6 Institute for Biophysical Dynamics, University of Chicago, Chicago, IL USA; 7 Present Address: Gene Center and Department of Biochemistry, Ludwig Maximilian University of Munich, Munich, Germany; 8 Present Address: Department of Biochemistry, University of Wisconsin Madison, Madison, WI USA; 9 Present Address: Takeda San Diego Inc., San Diego, CA USA; 10 Present Address: Division of Chemistry and Chemical Engineering, California Institute of Technology, Pasadena, CA USA

**Keywords:** Cryoelectron microscopy, X-ray crystallography

## Abstract

We propose the concept of universal fiducials based on a set of pre-made semi-synthetic antibodies (sABs) generated by customized phage display selections against the fusion protein BRIL, an engineered variant of apocytochrome b562a. These sABs can bind to BRIL fused either into the loops or termini of different GPCRs, ion channels, receptors and transporters without disrupting their structure. A crystal structure of BRIL in complex with an affinity-matured sAB (BAG2) that bound to all systems tested delineates the footprint of interaction. Negative stain and cryoEM data of several examples of BRIL-membrane protein chimera highlight the effectiveness of the sABs as universal fiducial marks. Taken together with a cryoEM structure of sAB bound human nicotinic acetylcholine receptor, this work demonstrates that these anti-BRIL sABs can greatly enhance the particle properties leading to improved cryoEM outcomes, especially for challenging membrane proteins.

## Abstract (web summary)

Single particle cryo-electron microscopy of membrane proteins is limited by their small size and difficulty to orient. Here the authors generate recombinant antibodies against the 12 kDa fusion partner BRIL domain from apocytochrome b562 to use them as plug and play fiducial marks for structure determination of BRIL fused membrane proteins.

## Introduction

Continuing technical developments have fueled transformative changes in the capability of determining biomolecular structures by both X-ray crystallography and single-particle cryo-electron microscopy (SP cryoEM). Structural biology problems that only a decade ago were deemed too daunting to even contemplate are now being routinely solved. One area that has been particularly affected is in the structure determination of membrane proteins. While X-ray crystallography remains a key player, it is the advent of SP cryoEM that has had the greatest impact^1–4^. However, despite many advantages, SP cryoEM currently has a serious blind spot—even with the recent advances, high-resolution structures of non-symmetric macromolecules are mostly limited to particles that exceed ~100 kDa in size^5,6^. Unfortunately, membrane proteins of therapeutic potential are, by and large, much below this threshold. For instance, molecular systems like GPCRs, ion channels and transporters are generally in the 50 kDa range or smaller. Further, membrane proteins are usually embedded in detergent micelles or nanodiscs making it even more difficult to accurately orient them.

We and others have shown that antibodies (Fabs) can be powerfully used as fiducial marks by adding mass (50 kDa) to the particle and assisting in its orientation^6–12^. Fabs have been generated by traditional hybridoma approaches, but generally recombinant-based library display methods, such as phage, yeast or ribosome display are more broadly used^13–15^. These display methods allow for the flexibility to tune affinities, epitopes and conformational state^16,17^. However, the use of Fabs remains restricted because they have to be produced for each individual system. While the display methods are in common practice, they are not yet turnkey for generation of high-affinity binders of the type needed for structural analyses, especially for membrane proteins.

To overcome these shortcomings, we endeavored to develop an approach that eliminates the need to generate Fabs for each system being studied. This approach exploits the concept of recombinant fusion proteins that have been employed to facilitate crystallization of membrane proteins. These fusion proteins are small stable proteins or domains that are introduced into the exposed loops of membrane proteins to provide additional lattice contact points for crystallization, as in the case of GPCRs^18^. We envisioned that these inserted modules would similarly prove to be very good epitopes for directed Fab binding. Further, a cohort of module-specific binders would obviate the need to generate protein-specific Fabs, thus providing “off-the-shelf” Fabs for use in structural studies.

However, turning theory into practice requires overcoming some inherent issues—notably the construction of the fusion partner to facilitate spatial compatibility while preserving its native structure. We had previously shown the effectiveness in using a Fab targeted to a portable RNA motif as a general RNA crystallization chaperone^19^. This work demonstrated the necessity of the inserted motif being structurally compatible with the target and anchored through it in a rigid fashion. Notably, this requirement of the rigidity of the connection between module and the protein of interest is even higher for SP cryoEM. The attributes of the Fab for orientation are directly related to its order with respect to the target; if the Fab is connected to a less ordered part of the molecule, this will greatly diminish its contributions to structure determination.

Guided by these principles, we focused on identifying and characterizing a module that could be seamlessly engineered into transmembrane α-helices (TMH), since TMHs represent the most prevalent type of transmembrane structural organization in many types of membrane proteins. While several fusion partners have been used for successful structure determinations of membrane proteins, we chose an engineered variant of apocytochrome b562, BRIL, that has a rigid helical domain making it an attractive partner for fusions mostly in the loops connecting α-helices of membrane proteins^18^ and also less frequently in the N- and C-termini^20,21^ leading to enhanced crystallizability of these targets. The 12 kDa BRIL has a 4-helix bundle fold arranged with the N- and C- termini separated by a distance similar to that between adjacent TMHs of α-helical membrane proteins. This arrangement facilitates its introduction in place of the intracellular loop-3 (ICL3) between helix V and VI of several GPCRs, without any disruption to the core structure of the GPCR^22–24^. However, small domains/proteins the size of BRIL, by themselves have insufficient mass to be effective fiducial markers^25^, especially for proteins of the size of GPCRs, transporters and heteroligomeric ion channels whose few recognizable extra-membranous features are masked by their solubilization medium, be it a detergent micelle, a nanodisc or an amphipol. We hypothesized that the addition of a 50 kDa Fab, with its distinctive elongated two-domain shape, would facilitate accurate orientation of the proteins, even when most of their structure is embedded in the membrane.

Herein, we describe the use of a semi-synthetic phage display library of humanized Fab fragments^26^ to generate a cohort of high-affinity synthetic antibodies (sABs) that bind to two distinct non-overlapping epitopes on BRIL. We show that the sABs bind to BRIL modules that have been engineered into several systems other than GPCRs. BRIL can be either inserted into a loop or added to the N- or C- termini of the protein. The binding and the effective rigidity of different sABs can be affected depending on the structural context of the inserted module. We show that it is relatively straightforward to alter the helical length between the target protein’s TMHs and the BRIL 4-helix bundle core resulting in the ability to modulate the sAB binding in a controlled way. Among the cohort of sABs, we identified sAB24 that was virtually universal—it bound under every manifestation of the BRIL incorporation. We affinity matured this sAB and obtained several variants with subnanomolar binding affinity to BRIL. The crystal structure of one of these variants, BAG2 bound to BRIL was determined at 1.9 Å. We determined the cryoEM structure of the pentameric, human α4β2 nicotinic acetylcholine receptor at 3.7 Å employing the variant BAK5 as a fiducial mark. Use of this sAB improved the overall resolution of the structure by breaking the pseudosymmetry during particle alignment and enabled us to distinguish between the different subunits in the receptor, which are virtually indistinguishable at low resolution. We believe that with these affinity-matured sAB variants, it will be possible for structural biologists to produce a highly effective 50 kDa fiducial mark to many membrane proteins in a “plug and play” fashion. This will open up new possibilities to more rapid determination of structures of multiple classes of important membrane proteins that heretofore required exceptional effort to determine, if they could be determined at all.

## Results

### Generation and characterization of sABs against BRIL

**Fig. 1:**
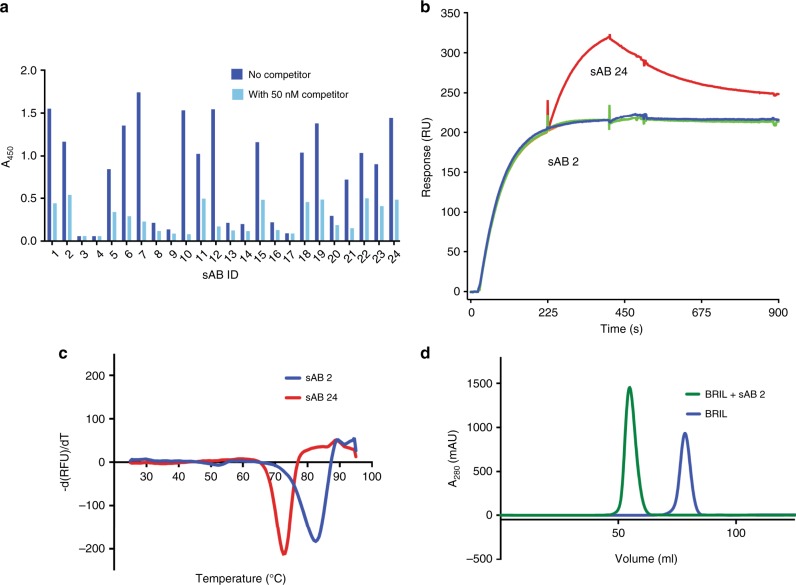
Characterization of the sABs against BRIL. **a** Representative single-point competitive phage ELISA of the binders obtained from phage display. Clones with strong ELISA signal in absence of competitor (dark blue bar) and good competition ratio are chosen for subsequent characterization. Low ELISA signal in presence of the competitor in solution (light blue bar) is a measure of good competition ratio. **b** Representative epitope binning by SPR shows that sAB2 (blue curve) and sAB24 (red curve) bind to two different epitopes. Most of the sABs share identical or partial footprints with sAB2. One such sAB sharing the same footprint with sAB2 is shown in green curve. **c** Thermal melting curves of sAB2 and sAB24 as monitored by DSF. **d** aSEC profiles show that the sABs form monodispersed complexes with BRIL.

In the phage display selection, we utilized a high-diversity (~10^10^) synthetic phage display library based on a humanized antibody Fab scaffold. To increase stringency, the selection was performed by decreasing the concentration of BRIL in each round. After four rounds of selection, 96 binders were screened using a single-point competitive phage ELISA (Fig. 1a). The clones that showed high ELISA signal and specificity were sequenced, resulting in 26 unique binders with high diversity in CDR-H3 and L3 indicating that they represented a large unique pool. These phagemids were sub-cloned into Fab format and were expressed and purified for further characterization.

To assess the diversity of the epitope space of the selected sABs, we performed epitope binning experiments using surface plasmon resonance (SPR). The binning experiments revealed that the sABs populated two distinct epitopes. The distribution of the two populations was surprisingly asymmetric. Epitope 1 is highly immunodominant, as 25 binders were directed towards it compared to only one sAB (sAB24) having a non-competing footprint with the first set (Fig. 1b). We then characterized the binding kinetics of seven sABs from Epitope 1 along with sAB24 from Epitope 2 (Supplementary Table 1). The binding constants (K_D_) for these sABs range are in low nanomolar (nM) range. The high affinity of these binders is principally due to a slow k_off_. The generated sABs are all highly thermally stable having melting temperatures (T_m_) above 70 °C (Fig. 1c). They also form monodispersed complexes with BRIL that can be isolated by size exclusion chromatography (SEC) (Fig. 1d).

### sAB24 binds to BRIL–GPCR chimeras in different conditions

**Fig. 2:**
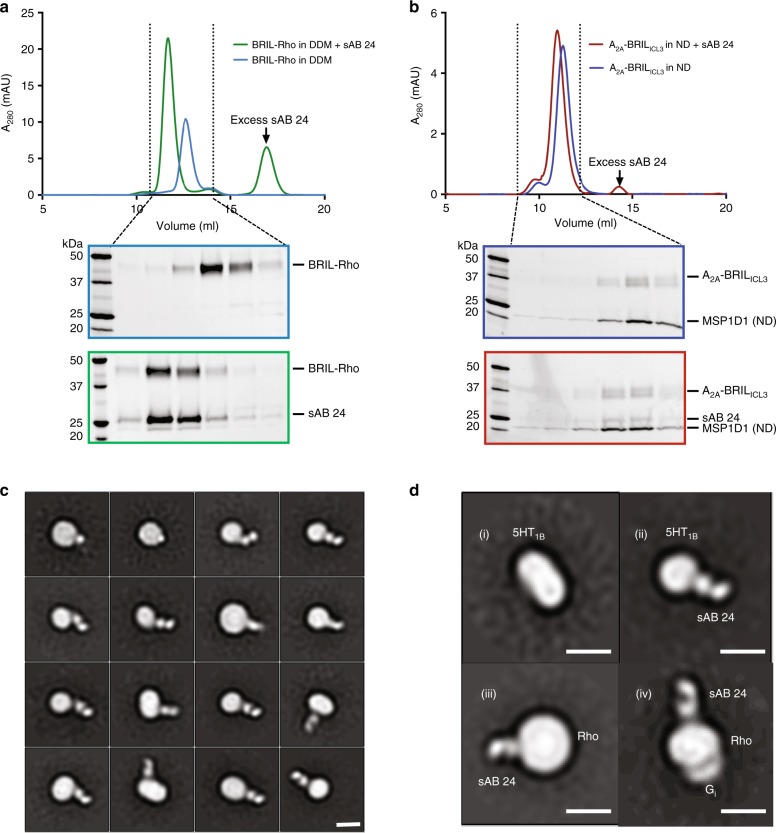
sAB24 binds to membrane proteins in detergents, amphipols, and nanodiscs. aSEC profiles and SDS-PAGE gels of complex formation of sAB24 with **a** BRIL-Rhodopsin in DDM and **b** A_2A_-BRIL in nanodisc 1D1. **c** 2D class averages from negative-stain images of sAB24 bound to BRIL-Rhodopsin in amphipol. **d** 2D class averages of the negative-stain images from: (i) BRIL-fused serotonin receptor (5HT_1B_) without sAB24 and (ii) with sAB24 in nanodisc E3D1, (iii) complex of BRIL-Rhodopsin and sAB24 in DDM (iv) complex of BRIL (N-term)-Rhodopsin bound to trimeric G_i_ and sAB24 in nanodisc E3D1. BRIL is fused to the N-term of Rhodopsin while the 3rd intracellular loop (ICL3) of the adenosine receptor A_2A_ and serotonin receptor 5HT_1B_ are replaced by BRIL. Micrograph of 5HT_1B_–sAB24 complex is in Supp. Figure 4a. Scale bars in (**c**, **d**) are 100 nm.

After initial characterization of the sABs, we tested their binding to GPCRs fused to BRIL either at the N-terminal or in place of ICL3 connecting helices 5 and 6. For the N-terminal fusion, we used rhodopsin as the model system, while the BRIL replacing ICL3 was tested on the adenosine receptor A_2A_ and serotonin receptor 5HT_1B_. aSEC experiments were performed with these constructs in detergent, nanodiscs and amphipols. In all cases, we were able to isolate the monodispersed complex from SEC. Representative chromatograms (Fig. 2a, b) and 2D class averages from negative stain (NS) (Fig. 2c) indicate the versatility of sAB24 under different experimental conditions.

Figure 2d(i) is a class average of the 5HT_1B_-BRIL(ICL3) insertion embedded in a nanodisc. BRIL is virtually imperceptible in the image, but with the addition of the sAB24, the image (Fig. 2d(ii)) shows a distinct appendage extending from the embedded protein that has a canonical two-lobe structure representative of the variable and constant domains of the sAB. Incorporating BRIL as an extension of N- or C-terminal α-helices is appealing because there are fewer restrictions compared to introducing BRIL replacing loops. However, if not properly designed, there might be a higher degree of flexibility at the ends of helices than when BRIL is constrained in a loop. Introducing BRIL on the N-terminal α-helix of rhodopsin suggests that in some circumstances a suitable fiducial can be produced by this strategy (Fig. 2d (iii)). This is also shown with the N-terminal BRIL introduced into rhodopsin bound to trimeric G_i_ ((Fig. 2d (iv)). In both these cases, the distinct bimolecular shape of sAB24 bound to the N-terminal BRIL is evident.

### Affinity maturation of sAB24

Although sAB24 has a relatively high affinity (K_D_ ~23 nM), the binding kinetics data indicated that its off-rate was not optimal for its use as a fiducial mark. Thus, we undertook an affinity maturation campaign that involved modifying the six CDR-H1 residues that were rich in Ser (^32^FSSSSI^37^). Our strategy was to design an affinity maturation library that introduced higher diversity into CDR-H1 in a way to bias the mutable positions to hydrophobic and aromatic residues. Using a combination of “hard” and “tailored” randomization strategies, the obtained phage library has a diversity ~10^10^ that ensures that all the generated mutants have been sampled several times in the pool.

**Fig. 3:**
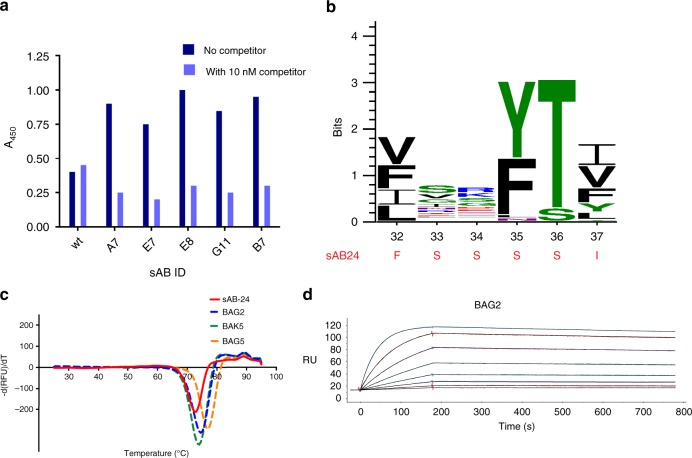
Characterization of the affinity-matured variants. **a** Competitive phage ELISA shows that the representative affinity-matured variants have a stronger ELISA signal (dark blue bar) and better competition ratio (light blue bar) than the wt sAB24 indicating a higher affinity compared to wt sAB. **b** WebLogo plot of the affinity-matured variants show that the positions 35, 36 in CDR-H1 are biased in aromatic (F/Y) and polar (S/T) aa residues. The CDR-H1 sequence of the wt sAB24 is highlighted in red. **c** DSF data show that the melting temperature of the wt and the affinity-matured variants are comparable. **d** SPR sensograms depicting kinetics of binding of BAG2 with BRIL. The sensograms show a slow rate of dissociation.

Selections were performed using a selection pressure to isolate binders with slower off-rates. Ninety-six clones were evaluated by single-point competitive phage ELISA, from which ten clones showed higher ELISA signal and competition ratio with respect to the initial sAB24 (Fig. 3a). The sequence alignment of the seven most promising affinity-matured variants (based on the ELISA signal and the competition ratio in phage ELISA) shows that there is a strong preference for aromatic residues (Phe, Tyr) at position 35 and polar residues, mainly Thr and Ser at position 36 (Fig. 3b). Among these seven variants, three were poorly expressed (<0.2 mg/L) and were subsequently triaged. The T_m_ values of the remaining variants all ranged between 74 and 77 °C (Fig. 3c). This indicates that the randomization strategy did not adversely affect the thermal stability of the sABs.

The binding kinetics of the affinity-matured variants show an increase in affinity between 50–150-fold compared to the wt sAB24 (Supplementary Table 2, Fig. 3d) that can be attributed both to the enhanced on-rate and slower off-rate for all the three variants (k_off_ ~0.9–2.0 × 10^−4^ s^−1^). While these results indicated that all the variants should be more potent as fiducial marks than the wild-type sAB, we selected the best expressing variant to determine which mutations in the CDR-H1 loop conferred high affinity to this variant. The SPR data indicated that BAG2 has a K_D_ ~0.3 nM. Ala scanning was performed on the six CDR-H1 residues (^32^VVDFSL^37^). Little difference in affinity is seen for Ala mutations at V32, V33, D34, and L37. However, the affinities of F35A and S36A decreased significantly with respect to BAG2 (Supplementary Table 3). The K_D_ values of F35A and S36A variants are 7.6 and 5.3 nM indicating a decrease in binding energy of ~2 kcal mol^−1^. For these two variants, the affinities were compromised by both a decrease in k_on_ and an increase in k_off_.

### Crystal structure of BRIL–BAG2 complex

**Fig. 4:**
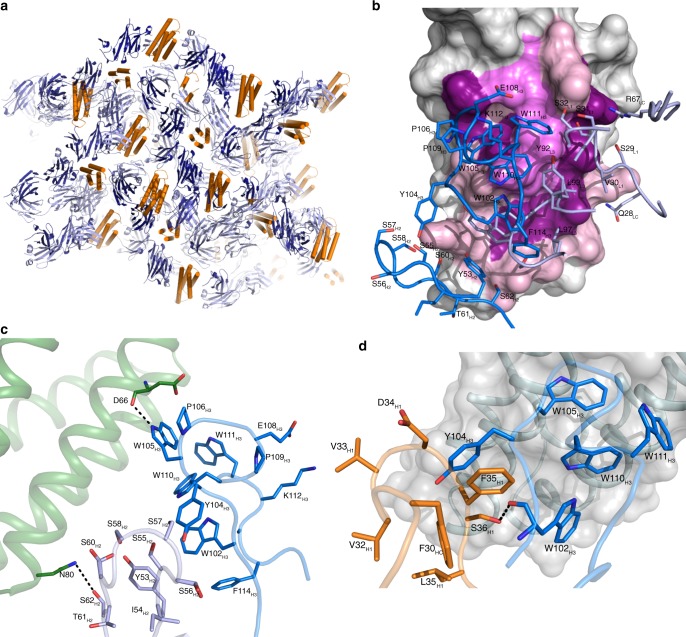
Structural characterization of the BRIL–BAG2 complex. **a** Analysis of the crystal packing shows the contacts in the crystal lattice are mediated primarily by BRIL and the heavy chain (HC) and light chain (LC) of BAG2. BRIL is colored orange while the HC and LC of BAG2 are colored dark and light blue, respectively. **b** Detailed picture of BAG2 residues (shown in sticks) interacting with BRIL. The HC and LC CDRs are colored dark blue and light blue respectively. Residues of BRIL were colored according to their percentage of reduction in accessible surface area upon complex formation (10–49%, light pink; 50–70%, pink; >70%, magenta). **c** Interacting residues of CDR–H2 (light blue) CDR-H3 (dark blue) of BAG2. Selected H-bonding interactions are highlighted. **d** F35_H1_ is involved in a π-stacking interaction with F30_HC_ and Y104_H3_ while hydroxyl group of S36_H1_ is hydrogen bonded with the main chain carbonyl oxygen of W102_H3_ in the crystal structure. These interactions demonstrate the conservation of the aromatic residues and Ser/Thr in positions 35 and 36 of CDR-H1, respectively, in all the affinity-matured variants (Weblogo Plot in Fig. 3b). Subscripts H1, H2, H3, L1, L2, L3 denote the CDRs and subscripts HC and LC denote the scaffold—e.g., F35_H1_ is a residue in CDR H1 while F30_HC_ is a residue in the HC scaffold, not in the CDR.

To delineate the interaction of the affinity-matured variant with BRIL, we determined the crystal structure of BRIL–BAG2 at 1.9 Å (Supplementary Table 4). The crystal contacts are mediated primarily by BRIL and the heavy chain (HC) and light chain (LC) of the sAB (Fig. 4a). BAG2 interacts with BRIL through both its HC and LC CDRs resulting in burial of 727 Å^2^ (HC (291 Å^2^), LC (436 Å^2^)) upon complex formation (Fig. 4b). This interface involves extensive hydrophobic, electrostatic, and hydrogen bonding interactions (Supplementary Table 5). Surprisingly, the LC CDRs interact somewhat more extensively with BRIL than the HC CDRs. Unlike CDR-H1, both CDR-H2 and CDR-H3 of HC interact with BRIL with the most extensive interaction mediated through the 13 residues long CDR-H3 loop, which is enriched in aromatic amino acids (Fig. 4c).

Interestingly, in the crystal structure, the mutated residues in CDR-H1 did not make any direct contact with BRIL itself, but appear to function through a series of indirect effects affecting the orientation of the CDR-H3. F35_H1_ and S36_H1_ showed drastic decreases in affinity when mutated to alanine. F35_H1_ is involved in an extensive π-stacking interaction with F30_HC_ and Y104_H3_ (Fig. 4d). We postulate that this interaction maintains the CDR-H3 in an optimized orientation that enhances the interaction of the CDR3 with BRIL resulting in the increased affinity of BAG2. Thus the position 35_H1_ is always enriched with aromatic residues, particularly Phe and Tyr after affinity maturation (Fig. 3b). The side chain hydroxyl group of S36_H1_ is hydrogen bonded with the carbonyl oxygen of W102_H3_ (Fig. 4d). Thus, 36_H1_ was always enriched with Ser and Thr although the library consisted of diverse residues for this position.

We superposed the crystal structure of the BRIL bound BAG2 on to the different published structures of GPCRs with BRIL fusions (either at the termini or ICL3) to analyze whether BAG2 will be useful as a versatile crystallographic/cryoEM chaperone for BRIL-fused targets (Supplementary Fig. 1). The alignments show no apparent clashes. This implies that the epitope of BAG2 should not be occluded in BRIL-fused constructs of GPCRs and thus, it has the potential to be used as a chaperone in structure determination of BRIL-fused GPCRs either by crystallography or by SP cryoEM.

### Portability of BRIL fusion to different types of membrane proteins

To gauge the potential versatility of the approach for membrane proteins other than GPCRs, we fused BRIL to: (i) The Zinc transporter YiiP—where BRIL was inserted by replacing the loop connecting the two helices 1 and 2 and helices 5 and 6 separately; and (ii) The potassium channel KcsA—where the N-term helix (1st 23 aa residues) was replaced by BRIL.

**Fig. 5:**
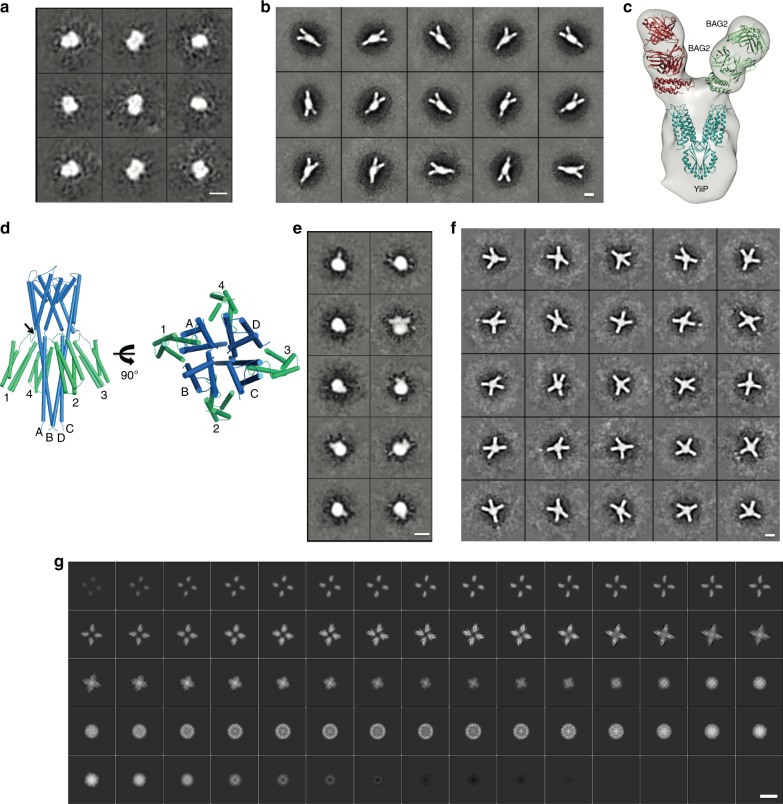
BAG2 binds to BRIL-fused constructs of different membrane proteins. 2D class averages from negative-stain images of **a** the zinc transporter YiiP-BRIL alone. **b** YiiP-BRIL bound to BAG2. Micrographs of YiiP-BRIL bound to BAG2 is shown in Supplementary Fig. 4b. **c** Three-dimensional map of YiiP-BRIL-BAG2 complex generated in cryoSPARC from the 2D class averages in (b). Crystal structures of YiiP (colored teal, PDB ID: 3H90) and BRIL–BAG2 (two copies colored red and green respectively, PDB ID: 6CBV) were fitted into the low-resolution map using Chimera. **d** Model of BRIL-(linker)-KcsA. The N-term helix of KcsA (aa residues: 1–23) was replaced with BRIL. The point of attachment is marked with an arrow. 4 copies of BRIL (labeled 1, 2, 3, 4; colored green) are fused to four copies of KcsA (labeled A, B, C, D; colored teal). Different helical linkers to fuse BRIL with KcsA were tested. 2D class averages from negative-stain images of **e** BRIL-2turn-KcsA alone **f** BRIL-2turn-KcsA bound to BAG2. Associated micrographs are in Supp. Figure 4c. **g** Volume slices through 3D reconstruction of the BRIL-3turn-KcsA/BAG2 complex from the cryoEM data. All scale bars in this figure are 100 nm. Representative cryoEM micrograph of the complex is in Supplementary Fig. 4d.

To introduce BRIL into dimeric YiiP, two different loops connecting helices 1–2 (residues 26–41) and helices 5–6 (residues 158–179) were assessed (Supplementary Fig. 2a). While the construct designed by inserting BRIL between helices 5–6 did not express, BRIL insertion between helices 1–2 increased the expression over 10-fold and the T_m_ of the BRIL-fused YiiP was 10 °C higher than the wt (Supplementary Fig. 2b). The 2D class averages from NS of YiiP-BRIL fusion alone (Fig. 5a) have no distinguishing features to suggest its orientation while that of the YiiP-BRIL/BAG2 complex show a dimeric arrangement of the sAB binding providing a clear guide towards the stoichiometry and the orientation of the particle (Fig. 5b, c).

To evaluate the extent and limitations of N- and C- terminal BRIL constructs other than GPCRs, we introduced BRIL into KcsA, a small tetrameric ion channel protein. KcsA has a N-terminal α-helix that lies parallel to the membrane on the cytoplasmic side and is linked to its first TMH. We substituted this N-terminal helix (1–23) with BRIL in a way where it could be systematically manipulated by adding residues to extend and rotate the module (Fig. 5d). BRIL was fused: (1) directly to the 24th residue of KcsA without any linker and via (2) three residues (-AAA-), (3) four residues or 1 turn (-AAAA-), (4) five residues (-AAAAA-), (5) six residues (-AARAAA-), (6) seven residues (-AARAAAA-), (7) eight residues or 2 turn (-AARAFAAA-), and (8) 11 residues or 3 turn (-EAAAKEAAAKA-) linker. In these constructs the helical structure was not only extended in a defined direction, but by increasing the linker length by one residue, the relative position of BRIL with respect to KcsA was also gradually rotated. Construct 1 disrupted the tetrameric assembly of KcsA, and thus was not pursued further. All the other seven constructs retained the tetrameric assembly of the channel. Regardless of the type of linker tested, four copies of BAG2 bind to all the constructs as determined by aSEC experiments (Supplementary Fig. 3). NS class averages of the construct with 2-turn linker clearly prove the utility of BAG2 as a fiducial mark. The class averages of the construct without BAG2 look like “blobs” (Fig. 5e) while the distinct tetrameric assembly of the complex is visible (Fig. 5f) after adding the sAB.

For further characterization, a small, preliminary cryoEM dataset was collected on the BRIL-3 turn-KcsA/BAG2 complex. We chose the longest (3-turn) linker in order to evaluate to what extent these connecting helical linkers can be used without introducing flexibility at the fusion sites. Volume slices through the 3D reconstruction clearly shows the secondary structural features for BRIL/BAG2 and the KcsA 4-helix bundle (Fig. 5g). While the 60 kDa KcsA will be a challenging target for structure determination by cryoEM, adding four copies of BAG2 to the target via BRIL makes the task much simpler by not only adding a mass of ~200 kDa to the complex, but also facilitating particle orientation by virtue of the distinct shape of the sAB so that even a partial dataset was able to discern the structural features of the target. This unambiguously demonstrates the potential of BAG2 as an effective fiducial mark when used in conjunction with a properly designed BRIL-fusion construct even at the N-/C-termini.

### sAB breaks pseuodsymmetry in heteropentameric ion channels

The cation-selective nicotinic acetylcholine receptor (nAChR) is a prototypical member of neurotransmitter-gated ion channels of the Cys-loop receptor family. The human α4β2 subtype is a heteropentamer and the α4, β2 subunits can assemble heterogeneously (2:3 or 3:2), which have nearly identical secondary structures. This hetero-oligomerization creates pseudosymmetry that makes the subunits indistinguishable at low resolution and thus, adds to the challenge of particle assignment in low-resolution cryoEM maps. To overcome this challenge, we explored use the anti-BRIL sAB as a fiducial mark for subunit identification and determination of stoichiometry, enabling reconstruction by cryoEM.

Different fusion constructs were designed by inserting BRIL in the α4 or β2 subunits in the region of the loop deletions between helices MX and M4 (α4—deleted loop residues 339–555, β2—deleted loop residues 327–416). The boundaries were varied on the C-terminal side of BRIL by 7 aa residues into the receptor subunit, (Supplementary Table. 6a and Supplementary Fig. 5a, b). BRIL fusions in α4 were partnered with the β2 construct, while BRIL fusions in β2 were partnered with the α4 construct (Supplementary Table 6b). Small scale expression of these constructs in the presence of nicotine was followed by quantitation of receptor at the cell surface using a Guava assay (Supplementary Fig. 5d) as detailed in the Methods. The constructs with poor cell surface receptor expression were discarded (Supplementary Table 6b) and the best expresser for each class of fusion protein was carried forward for large scale expression and purification. After evaluation by aSEC, the constructs displaying a homogenous receptor peak, with minimal aggregation, were selected for formation of a binary complex with another affinity-matured variant BAK5. Construct no: 8 with BRIL fused in the α4 subunit was the best behaved and SDS-PAGE analysis of the construct showed that the ratio of α4:β2 in the receptor is 2:3 (Supplementary Fig. 5e). The monodisperse complex with BAK5 was isolated from aSEC and subjected to NS to confirm the subunit stoichiometry of the sAB bound receptor. For cryoEM sample preparation, the expression was repeated for this selected construct in the presence of the partial agonist varenicline, purified to homogeneity as a complex with BAK5 and vitrified for data collection.

**Fig. 6:**
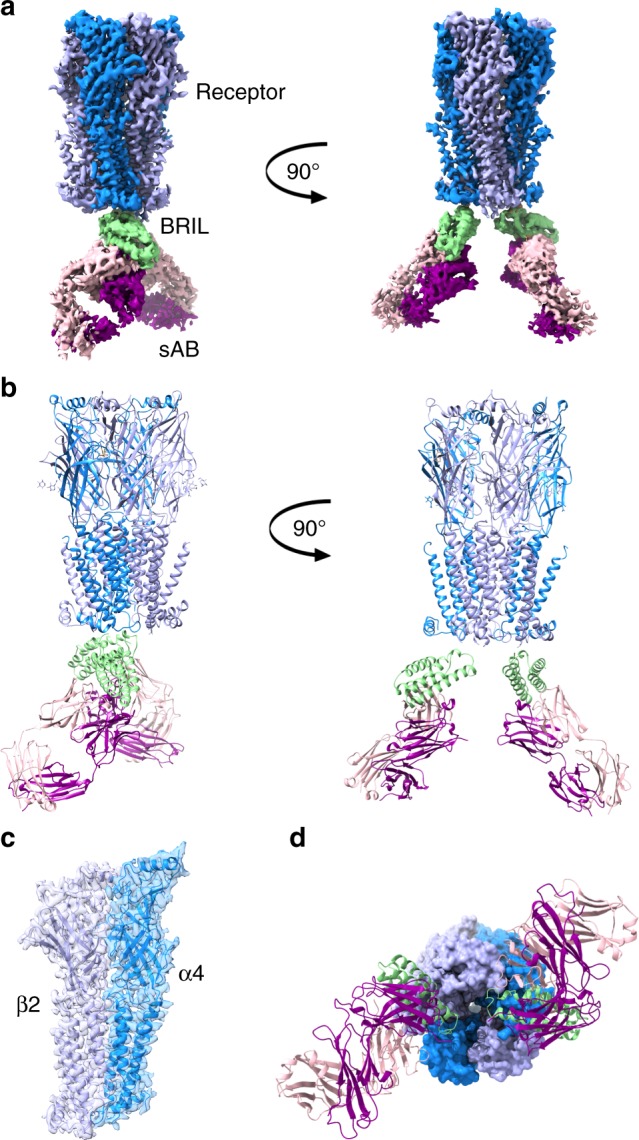
CryoEM structure of the human α4(BRIL)β2 nicotinic acetylcholine receptor bound to sAB BAK5. **a** EM density map of the entire complex. **b** Cartoon representation of the receptor fused to BRIL in complex with the sAB. **c** Well defined electron density map of the α4 and the β2 subunits of the receptor at the interface. **d** Bottom-up view of the complex. In all the panels, the α4 and the β2 subunits of the receptor are colored marine and light blue, respectively, BRIL is colored green, the light chain (LC) and heavy chain (HC) of the sAB is colored light pink and magenta, respectively.

**Fig. 7:**
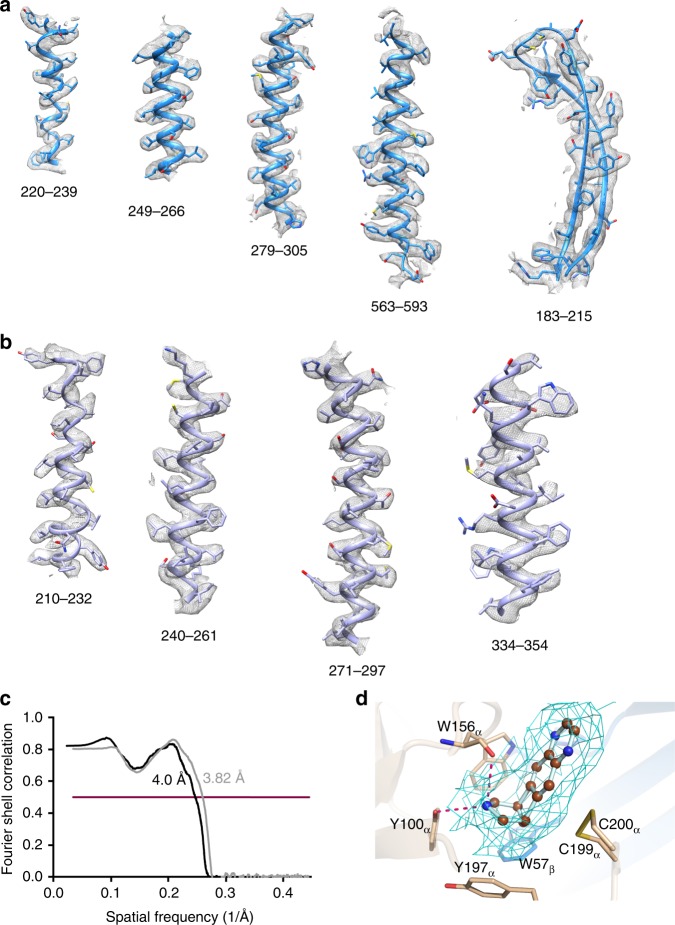
Model-map validation. Secondary structure elements of a representative **a** α4 subunit and **b** β2 subunit are shown fitted to the cryoEM maps **c** Fourier shell correlation (FSC) curve for the model vs map focused on α4β2 receptor (gray) and vs map of α4β2:sAB complex (black) with indicated resolutions at FSC = 0.5. **d** CryoEM map (mesh) density accounting for varenicline in the binding pocket. Selected residues on β2 subunit (marine) and on α4 subunit (wheat) in contact with the compound are shown. H-bonding interactions between the piperidine nitrogen of varenicline and the aa residues are highlighted in red.

We determined the cryoEM structures of the sAB bound receptor at a global resolution of 3.7 Å and 3.9 Å (Supplementary Table 7 and Supplementary Figs. 6, 7). The two copies of sAB bound to the two α4 subunits fused to BRIL were readily distinguishable indicating that the module was introduced without disrupting the structure of the subunit (Fig. 6). Local resolution at the core of the receptor is ~3.5 Å (Supplementary Fig. 8) and is consistent with the side chain features throughout the density maps (Fig. 7a, b). Additionally, the EM density corresponding to varenicline was clear in the classical neurotransmitter site at the α–β interface as seen in acetylcholine-binding proteins from *Capitella teleta* and *Aplysia californica* and 5-HT binding protein^27–29^. Varenicline is stabilized through hydrophobic interactions with the aromatic residues as well as the vicinal disulfide-bonded Cys199 and Cys200. The piperidine nitrogen is positioned to form hydrogen bonds with the phenolic hydroxyl group of Tyr100 as well as with the Trp156 backbone carbonyl. (Fig. 7d). BAK5 bound to the BRIL-fused α4 subunit broke the pentameric pseudosymmetry of the molecule, thereby enabling facile particle orientation during data processing and reconstruction. Insertion of BRIL did not introduce any local or global distortions in the structure as evident by superposition of the BRIL-fused α4 subunits with the α4 subunits from PDB ID: CNJ^12^ without any BRIL fusion (RMSD C_α_ = 0.7 Å) (Supplementary Fig. 9). This is a clear demonstration that this strategy can be employed in similar situations with BRIL-fused constructs of other targets, establishing the sAB as a “universal fiducial mark”.

## Discussion

Fab fragments have been extensively used as crystallization chaperones as they bind to the target protein to provide homogeneous and stable surfaces to promote lattice contact points^30^. More recently, Fabs have taken on an increasingly important role in SP cryoEM studies as fiducial marks due to their size (50 kDa), shape, and rigid structure. They appear as an appendage of a length of about 70 Å extending from the target molecule and as such, provide a powerful guide point for accurate orientation of small particles. Notably, even at lower resolutions the variable and constant domains of Fabs usually form a recognizable bimolecular shape. From our experience, this feature is a good indicator of a quality fiducial mark that will help to facilitate a high-resolution structure determination. Interestingly, at higher resolution, the space between the variable and constant domains is readily discernable as a “hole”, adding another feature to assist in orientation of the particle.

Even considering all their attributes, the use of Fabs for structural biology applications is still limited because of the requirement that they need to be generated to the particular target system being studied. Kim et al. have proposed the use of anti-helix antibodies generated by “epitope transplant” to off-target proteins as versatile chaperones in structural biology^31^. However, this technology has its own set of challenges^32^. We endeavored an alternate strategy to develop a class of “universal” sABs that involves having one or a few sABs, which bind to a structural element in the form of a domain, or motif that is inserted into the protein of interest. As a consequence, if that structural element can be introduced without disrupting the protein’s structure, it should be possible to add such a sAB in a “plug and play” fashion to be utilized as a ready-made fiducial mark or crystallization chaperone.

Although this concept is quite simple, the ultimate usefulness of these chimeric constructs depends on quite specific structural and spatial conditions. A guiding principle in our design strategy was the requirement that the inserted domain be engaged rigidly to the structural framework of the parent protein. Demonstrably, Fabs attached to flexible entities have diminished potential as structural chaperones for either cryoEM or X-ray applications. With this in mind, we tested and characterized two types of design in our chimeric constructs. In one manifestation of our design, the 4-helix bundle BRIL is inserted into a surface loop following similar strategies developed for the GPCR examples involving insertion of BRIL into the ICL3 loop. Here BRIL is connected through its N- and C- terminal to form continuous extensions of two α-helices of the membrane protein. Thus, the length and orientation of the BRIL extension can be manipulated by adding full or partial turns to the helices. While optimization may be necessary, it should provide a rigid landing pad for Fab binding. This is demonstrated for sAB24 binding to a BRIL insertion into the ICL3 loop of the serotonin receptor 5HT_1B_, resulting in an easily recognizable fiducial for this small nanodisc embedded membrane protein. We also showed that this BRIL insertion strategy is portable to other proteins aside from GPCRs like the zinc transporter YiiP. Another important application of the loop-inserted BRIL domain is its utilization to distinguish between homologous subunits in hetero-subunit complexes as was the case for α4β2 nAChR. While nAChR is of sufficient mass (~310 kDa) for cyroEM analysis by itself, orientation of the particle is highly compromised, especially at low resolution since it is not possible to distinguish between the homologous subunit types or their stoichiometry. Thus, the previously determined α4β2 nAChR structure relied on generating antibody fragments to one of the subunit types by traditional approaches^12^. We demonstrated here that the process of generating a system specific Fab can be circumvented by inserting the BRIL domain into the α4 subunit, which breaks the pseudosymmetry, making orientation of the particle straightforward once the Fab is added. Notably, in concurrence with similar observations, small domains (<15 kDa) are not useful fiducials by themselves when inserted into large multimeric membrane proteins without adding additional mass to them^25^.

An alternate design exploits the fact that many targets, especially membrane proteins have α-helices at their N- or C- terminal ends. For this class of proteins, BRIL can be fused as a continuation of the helical structure at the appropriate end. The difference between the two strategies is that when BRIL replaces the ICL3, it is “pinned” into position at its two ends. It might be expected that fusing BRIL at the N- or C- termini of proteins could suffer from increased flexibility resulting in less effective fiducial marks. At some level, this is probably true; however, our negative-stain data suggest that the strategy of fusing BRIL to the termini of proteins through α-helical extension results in a surprisingly rigid structural construction. This premise is demonstrated by the negative staining class averages of BRIL fused to the N-terminus of rhodopsin. There is a clear distinction between the variable and constant domains of sAB24 where this bi-module feature is a hallmark of the sAB being highly ordered relative to the parent protein (Fig. 2d (iii), (iv)).

In another series of experiments testing an expanded spectrum of the terminal fusion strategy, we introduced BRIL into varying N-terminal constructs of KcsA. We inserted BRIL into the first transmembrane α-helix of the channel by replacing the first helix. By introducing alanines or a combination of alanines with other helix-philic residues at the juncture of the BRIL–TMH connection, we could effectively control the distance and orientation of the BRIL with respect to the membrane and the cytoplasmic portion of the channel. Simple modeling indicated that the BRIL is on the cytoplasmic side of the channel. One might picture a situation when the orientation of the BRIL would be such that the engaged sABs would adopt an outward pointing pattern, but given the size of the sABs (which are about the size of the channel itself) and the presence of the cytoplasmic portion of the channel, there might exist significant spatial limitations for how the sABs can engage BRIL in the context of the different constructs. Thus, it was surprising that virtually all constructs with BAG2 formed competent complexes. We interpret these results as the high-affinity interaction between BRIL and BAG2 overcoming the energy associated with steric crowding, allowing all four sABs to form the specific packing arrangements in all of the tested constructs. Importantly, the negative-stain class averages seem to indicate that the packing patterns still result in useful fiducials for particle alignment.

Currently, progress in determining SP cryoEM structures of smaller macromolecular systems is frustrated by both the technical challenges described above and the availability of state-of-the-art microscope time. This is very similar to the situation for X-ray structure determination several decades ago before the current generation of synchrotrons, detectors, computing and software that are now commonplace, were developed and implemented.

Fortunately, a similar evolution in instrumentation and related technologies is ongoing and will continue in the SP cryoEM field. This will inevitably lead to expanding the range of targets that can be accessed, as well as reducing the times for collecting and processing high quality data. However, tools like sAB-based crystallization chaperones and fiducial marks will always have an impact on improving the level of structural information extracted from these techniques. The technology advanced here is to enable structural biologists to exploit these sABs for cryoEM (or crystallography) without having to incorporate antibody engineering into their workflow. Only relatively straightforward molecular biology is required to introduce modules like BRIL into the protein of interest. Thus, once done, “off the shelf” sABs can be added to provide samples of sAB-target complexes ready for evaluation and analysis.

## Methods

### Phage display selection

To obtain sABs, a selection campaign was performed on biotinylated BRIL using library E. A cysteine mutation was introduced at the C-terminus of the wt BRIL for labeling. The cysteine mutant was chemically biotinylated specifically at the cysteine residue using EZ-link Maleimide PEG2 biotin (Pierce) and the labeling efficiency was tested using a streptavidin (SA) pull down assay. Panning was done at room temperature following published procedures^33^. In the first round, 1 ml of phage library E^26^ containing 10^12^–10^13^ virions was added to SA magnetic beads (Promega) precoated with 100 nM of biotinylated target. The phage pool was incubated with the beads for 30 mins. Beads containing bound virions after extensive washing were used to infect freshly grown log-phase *E. coli* XL1-Blue cells. Phages were amplified overnight in 2YT media with 50 μg/ml Amp and 10^9^ plaque-forming units/ml M13-KO7 helper phage. To increase the stringency of selection, three additional rounds of sorting were performed by decreasing the target concentration in each round (second round-50 nM; third round-10 nM; fourth round-10 nM), using the amplified pool of virions of the preceding round as the input. Sorting from the second to fourth rounds was done on a Kingfisher instrument. From the second round onward, 0.1 M glycine; pH 2.7 was used to elute the bound phage from the immobilized target. To eliminate the potential SA binders, the amplified phage pool after each round was negatively selected against SA beads before using as input for the next round. After four rounds of selection, clones with high binding affinity and good competition ratio were chosen based on single-point competitive phage ELISA and sequenced to identify unique binders. The unique binders were cloned in vector pRH2.2, expressed in *E.coli* BL21(gold) cells and purified following published protocols^34^.

### Kinetics and epitope binning by SPR

Interaction analyses between BRIL and sABs were performed at 20 °C using a BIACORE 3000 (GE Healthcare) by immobilizing His_10_-BRIL onto a nitrilotriacetic acid sensor chip via the N-terminal His_10_ tag. For each assay, 2-fold dilution series of sABs were injected at a flow rate of 30 μl/min. All conditions were tested for at least five different sAB concentrations. Sensograms were double-referenced using blank channel and buffer injections. Data processing and kinetic analysis were performed using BiaEvaluation software. To determine kinetic rate constants, all datasets were fit to a 1:1 interaction model.

For the epitope binning experiments, 100 nM sAB2 was injected on His_10_-BRIL immobilized on the sensor surface to saturate the epitope of interaction. This was followed by injecting a mixture of sAB2 and another sAB to be tested (100 nM each). 100 nM sAB2 was added in the second injection to make sure that the epitope of SAB2 remains fully saturated during the interaction of BRIL with the second sAB. A control experiment was performed using sAB2 alone in the second injection to make sure that the epitope of sAB2 on BRIL was saturated. Thus any increase in response units during second injection can be attributed to the second sAB binding to an epitope different from that recognized by sAB2.

### Generation of phage library for affinity maturation

For affinity maturation of CDR-H1, we used both “hard” and “tailored” randomization strategies simultaneously to create a phage library using the following oligo (P1) containing degenerate codons: 5′ gcagcttctggcttcaac NTT NNC NNS NWW NNT NTT cactgggtgcgtcag 3′(N = A/C/G/T, S = G/C, W = A/T). Stop codons were introduced in the CDR-H1 of wt phagemid (sAB24) by quick-change mutagenesis. ssDNA isolated from the phagemid containing the stop codons was used as the template for library generation by Kunkel mutagenesis^35^ with phosphorylated oligo P1 designed to simultaneously repair the stop codons and introduce mutations at the designed sites. The phage library was generated through electroporation of SS3320 cells^36^. Recovered cells were infected with M13-K07 helper phage and allowed to amplify overnight at 37 °C. Phage particles were isolated through PEG/NaCl precipitation for subsequent selection.

### Off-rate selection for affinity maturation

Three rounds of sorting were performed. To enhance the efficiency of affinity-based selection and enrich for binders with very slow off-rates, a solution-based sorting strategy using excess of non-biotinylated BRIL (nbt-BRIL) as competitor was adopted^37^. The concentration of biotinylated BRIL (bt-BRIL), the concentration of the competitor and the duration of the competition steps modulated stringency. In 1st round, 1 nM bt-BRIL was incubated with the phage library for an hour followed by incubation with 1uM nbt-BRIL for 15 min. This mixture was then bound to the SA magnetic beads for 10 mins, washed with PBST/0.5% BSA and eluted with 0.1 M glycine, pH 2.7. The eluted pool after neutralization with 1.0 M HEPES, pH 7.5 was used to infect log-phase XL1 cells for overnight amplification of phage. In the second round, 0.1 nM bt-BRIL was used followed by incubation with 100 nM nbt-BRIL for 30 min. The 3rd round used 0.1 nM target followed by incubation with 100 nM nbt-BRIL for 2 h. Amplified phage pool from elution of each round is used as the input in the next round.

### Expression and purification of the constructs

Wt and Cys mutant-BRIL: *E. coli* BL21 (DE3) was used for expression. Cells were grown in 2xYT media supplemented with kanamycin to OD_600_ = 0.6 and induced with 1 mM IPTG at 37 °C. Cells were harvested after 4 h, lysed by sonication and purified by Ni-NTA chromatography followed by TEV treatment to remove the His-tag and finally SEC on Superdex 75 column. For purification of the cysteine mutant of BRIL, the buffers were supplemented with 200 uM TCEP.

YiiP-BRIL: The YiiP-BRIL-fusion construct was expressed using the restrained expression method^38^. Briefly, *E. coli* BL21-AI cells (Invitrogen) were transformed with the plasmids. Cells were grown in TB media supplemented with 0.4% glycerol and kanamycin to OD_600_ = 0.6 at 37 °C, subsequently induced with 0.01% final concentration of arabinose, and allowed to express protein for 20–24 h at 16 °C. Harvested cells were lysed by sonication and cell debris were cleared by centrifugation at low speed (6000 × *g*). Cell membranes were isolated by ultracentrifugation (150,000 × *g*) for an hour. YiiP-BRIL was purified from isolated membranes using a previously described protocol^39^.

KcsA-BRIL: *E.coli* BL21(DE3)plysS cells were transformed with the KcsA-BRIL/pET28a plasmids. Cells were grown in 2xYT media supplemented with kanamycin to OD_600_ = 0.6 induced with 1 mM IPTG at 37 °C and harvested after 3 h. Harvested cells were resuspended in buffer A (50 mM HEPES; pH 7.5, 200 mM KCl) containing protease inhibitors, DNase and lysed by French press. Cell debris including membrane fractions were isolated by ultracentrifugation at 150,000 × g for 1 h. The pellet containing the membrane fraction obtained was solubilized by 1% DDM in 50 mM HEPES; pH 7.5, 200 mM KCl for 1 h at rt. The supernatant containing solubilized protein was separated from the cell debris by another round of ultracentrifugation at 150,000 × *g* for 1 h. The supernatant was incubated with 5 ml Talon resin for 1 h at rt. The resin was washed with buffer A containing 0.05% DDM and 10 mM imidazole. Protein was eluted with buffer A containing 0.05% DDM and 300 mM imidazole. The eluted protein was concentrated with an Amicon concentrator (MWCO = 100 kDa) and subjected to SEC on a Superdex 200 Increase column in buffer A with 0.05% DDM. Peak fractions containing aggregate free KcsA-BRIL were pooled and stored at 4 °C for further validation.

A_2A_-BRIL (ICL3): The receptor was expressed in *Sf*9 cells and purified following published protocol^22^.

BRIL-Rho: Isolated membranes from 1 L of *Sf9* cell culture were diluted to 50 ml in 20 mM HEPES (pH 7.4), 400 mM NaCl, 10% glycerol supplemented with Complete protease inhibitors (Roche). Detergent stock was added to final 0.5% DDM, 0.1% cholesteryl hemisuccinate (CHS) (Anatrace) and incubated for 3 h with gentle mixing. The sample was clarified by ultracentrifugation and incubated with 5.0 mL TALON resin overnight at 4 °C. The resin was washed with 20 CV of wash buffer (20 mM HEPES (pH 7.4), 400 mM NaCl, 10% glycerol, 0.03% DDM, 0.01% CHS, 25 mM imidazole). Protein was eluted in minimal volume with elution buffer (20 mM HEPES (pH 7.5), 400 mM NaCl, 10% glycerol 0.03% DDM, 0.01% CHS, 250 mM imidazole). Eluted sample was concentrated using Amicon concentrator (MWCO = 100 kDa) and loaded onto Superdex 200 Increase column pre-equilibrated in gel filtration buffer (20 mM HEPES (pH 7.4) 400 mM NaCl, 10% glycerol, 0.03% DDM, 0.01% CHS). Peak fractions were pooled and kept at 4 °C for further experiments.

5HT_1B_-BRIL (ICL3): The receptor was expressed in *Sf*9 cells and purified following published protocol^23^.

α4(BRIL)β2 nicotinic acetylcholine receptor (nAChR): The design of expression constructs and protein expression are as follows—all human α4 (Uniprot P43681) and β2 (Uniprot P17787) constructs were synthesized at Genewiz and sub-cloned into pcDNA3.1(+) (Life Technologies). They include the signal peptide and encode residues 1–338 and 556–601 of the α4 subunit, and residues 1–326 and 417–477 of the β2 subunit (numbering reflects mature protein after signal peptide cleavage). A Glu-Arg linker was inserted in the MX–M4 junction, between Phe559 and Ser560 in the α4 subunit and between Gln420 and Ser421 in the β2 subunit. For purification purposes, a Strep-tag was inserted at the C terminus of the β2 subunit preceded by a Ser-Ala linker. Additionally, BRIL-fusion constructs were designed (Supplementary Table 6) for both the α4 and β2 subunits. Endotoxin free plasmid DNA was generated for all constructs (Qiagen).

The receptor was expressed in Expi293F cells using the Expifectamine 293 Transfection kit protocol (Life Technologies). Constructs were first tested at small scale (60 mL) in the presence of nicotine and were triaged by cell surface expression using a guava assay (described below). To promote the stoichiometry of (2)α4:(3)β2, cells were transfected with ~1:2 ratio of α4 endotoxin-free plasmid to β2 endotoxin-free plasmid. The best expresser from each class of fusion protein was scaled-up to 1 liter where cells were transfected with 330 ug of α4 and 670 ug of β2 endotoxin-free plasmid. Nicotine (Sigma-Aldrich) or varenicline were added at the time of transfection at a final concentration of 100 uM. Cells were cultured at 37 °C for 18 h, transferred to 30 °C, then harvested 70 h post-transfection by centrifugation and were stored frozen at −80 °C. The expression of the receptor was monitored by Guava assay that is as follows—at the time of harvest, 10 uL of culture from transfected and non-transfected Expi293 cells were collected and transferred to 96-well plates and were incubated with 20 uL of either 1:100 StrepMAB-Classic Chromeo 488 conjugate, (IBA Lifesciences, 2-1544-050) or 1:100 Anti-FLAG-FITC antibody (Sigma, F4049), which had been diluted in 4% BSA in TBS plus 3 uL 7AAD (BD-Pharmingen, 559925). After incubation with gentle agitation at 4 °C for 20–30 min, each well was washed by addition of 200 uL of TBS, followed by centrifugation at 500 × *g* for 5 min. The supernatant was discarded and the cells were resuspended in 200  uL TBS. The samples were then analyzed using red (live/dead) and green fluorescence (non- expressing/expressing) on a Guava EasyCyte HT.

Transfected cells were thawed, resuspended with 50 mL of lysis buffer (20 mM Tris pH 7.5, 0.15 M NaCl, 1 mM TCEP, EDTA free protease inhibitor cocktail tablets (Roche), 100 uM Varenicline, and benzonase, lysed on ice using a microfluidizer and clarified by centrifugation at 50,000 × g for 1 hour at 4 °C. The pellet from 1 L of cells was resuspended in lysis buffer supplemented with 2% DDM, 0.01% CHS to solubilize protein at 4 °C for 1 hour. After centrifugation at 50,000 × g for 1 hour at 4 °C, the supernatant was loaded onto 5 mL of Streptactin Superflow Plus resin (Qiagen), which had been equilibrated in buffer containing 20 mM Tris pH 7.5, 0.15 M NaCl, 0.05% DDM, 0.01% CHS, 1 mM TCEP, EDTA free protease inhibitor tablets (Roche) and 100 uM varenicline. After washing to a stable baseline, bound protein was step eluted in equilibration buffer supplemented with 10 mM desthiobiotin. Fractions were analyzed by stain-free SDS-PAGE (BioRad) and pooled based on the presence of the α4 and β2 subunits (Supplementary Fig. 8a). The pool was treated with benzonase, supplemented with 15% glycerol, aliquoted, flash frozen in liquid nitrogen and stored at −80 °C.

### Thermal stability assays

Thermal stabilities of soluble proteins were measured by differential scanning fluorimetry (DSF)^40^. DSF measurements were performed on a Bio-Rad CFX384 real-time PCR instrument. Samples were prepared in triplicates in 50 mM sodium phosphate buffer (pH 7.4) with 150 mM sodium chloride and 4 × (250-fold dilution of 1000X stock) Sypro Orange dye to a total sample volume of 25 μl. Concentration of protein was 4 μM in samples containing either sAB or target. For target/sAB complexes, 4 μM target was mixed with 6 μM sAB in 1:1.5 molar ratio. Thermal melts were performed by heating the samples from 25 °C to 95 °C, increasing the temperature in steps of 0.5 °C/30 s. Wavelengths of 490 and 575 nm were used for excitation and emission, respectively. Obtained data were processed with the CFX software provided by the manufacturer. The first derivative of the curve is used to determine the melting temperature (T_m_).

The thermal stability of the membrane proteins (wt YiiP and the YiiP-BRIL chimera) was determined using the thiol-specific fluorochrome N-[4-(7-diethylamino-4-methyl-3-coumarinyl)phenyl]maleimide (CPM)^18^ in a HORIBA Fluorolog-3 fluorimeter.

### Reconstitutions of GPCRs in nanodiscs and amphipols

A_2A_-BRIL (ICL3) in nanodisc: Freshly purified receptor was directly used for nanodiscs incorporation. For incorporation 10 mM stock of lipid mix of 75% (3:1 molar ratio of POPG:POPC) and 25% CHS in 30 mM DDM was prepared. Purified receptor was mixed with lipid mix and incubated on ice for 20 min. Subsequently MSP1D1 protein was added and mixture was incubated on ice for 5 min. Reconstitution mixture was incubated with activated Bio-Beads SM2 at 4 °C overnight. The final ratio of receptor:MSP1D1:lipid was 1:10:550. For detergent removal 150 mg of Bio-Rad SM-2 absorbent beads was used per 1 mg of DDM. Nanodiscs with embedded receptor-BRIL chimera were separated by Ni-NTA chromatography followed by size exclusion chromatography on Superdex 200 Increase column pre-equilibrated in gel filtration buffer (25 mM HEPES (pH 7.4) 200 mM NaCl, 10% glycerol) and collected for complex formation with sABs.

BRIL-Rho in amphipol: 500 ul of 1 mg/ml protein in buffer containing 0.02% DDM was mixed with 20 ul of 10% amphipol A8-35 (Anatrace) and incubated for 30 min at 4 °C. Then 2 mg of Bio-Rad SM-2 absorbent beads was added to it and incubated at 4 °C for 4 h. The beads were removed and the supernatant loaded on S200 column equilibrated with buffer (20 mM HEPES, 100 mM NaCl, pH 7.4) without detergent to remove the free amphipol. The peak fractions were collected for forming complex with sABs.

5HT_1B_ and BRIL-Rho in nanodisc: Purified receptor was mixed with the soybean lipid stock and MSP1E2 at the ratio of 1:2:160 and incubated on ice for 30 min Bio-beads SM2 (200 mg per 1 ml of mixture), were added to initiate the reconstitution and the mixture incubated at 4 °C overnight. Bio-beads were then removed and the reconstitution mixture was loaded on a Superdex 200 column (GE) in buffer (20 mM HEPES, 100 mM NaCl, pH 7.4). The peak corresponding to 5HT_1B_-BRIL reconstituted in lipid nanodisc was collected for forming complex with sABs.

### Complex formation of BRIL-fused targets with sABs

Purified BRIL-fused targets in respective buffers were mixed with 1.2–1.5-fold molar excess of sABs and incubated on ice for 30 min. Subsequently they were run on Superdex 200 Increase/Superose 6 Increase columns (depending on the target) pre-equilibrated in sample specific buffers of choice. Complex formation was analyzed by changes in retention volume and protein gel electrophoresis.

### Crystallization and structure determination

A crystal structure of BRIL in complex with sAB BAG2 was determined. His_10_-wt BRIL was purified by Ni-NTA resin followed by treatment with TEV protease to cleave the His-tag. The protein was further purified on Ni-NTA resin to eliminate the tag and the protease. The flow-through fractions containing the tag free protein from the subtractive purification step were concentrated and further purified to monodispersity by SEC using a 16/60 Superdex 75 column on AKTA explorer. BRIL/BAG2 complexes were formed by incubating purified BAG2 with a 2-fold molar excess of monodispered BRIL. The 1:1 complex was purified from excess BRIL by SEC using a 16/60 Superdex 75 column equilibrated with 20 mM HEPES, pH 7.5, 150 mM NaCl. Crystallization trials were setup with the concentrated, monodispersed 1:1 BRIL/BAG2 complex at 40 mg/ml using PEG-ION suite from Hampton Research and NEXTAL Protein Complex Suite from Qiagen at 19 °C. Crystals appeared at multiple conditions in the screens. Crystals that grew from 0.2 M Sodium formate, 20% (w/v) PEG 3350 in PEG-ION screen were cryoprotected in 20% glycerol and flash-frozen in liquid nitrogen. Data were collected at 24-ID-C at the Advanced Photon Source and processed with XDS^41^. Initial phases were determined in Phaser^42^ by molecular replacement using previously determined crystal structures of BRIL (PDB code: 4EIY)^22^ and sAB (PDB code: 5BJZ)^17^ without CDRs as models. Pieces of variable and the constant domains of sABs were used as different ensembles during molecular replacement. The initial promising solution obtained from Phaser was subjected to iterative cycles of refinement in Phenix^43^ alternated with manual model building in Coot^44^ and addition of solvent molecules. The progress of the refinement was monitored by a steady decrease and convergence of R and R_free_ values. The stereochemical quality of the model was validated using Molprobity^45^. The data collection and refinement statistics are shown in Supplementary Table 4. Solvent-accessible surface areas and the pi-stacking interactions were calculated using PISA^46^ and RING server^47^ respectively. The figures were created in PyMOL^48^. The atomic coordinates and structure factors have been deposited in the Protein Data Bank (http://wwpdb.org/) with the PDB code 6CBV.

### Negative-stain analyses

BRIL-Rho and 5HT_1B_ receptor complexes: Protein samples were applied to a freshly glow-discharged carbon-coated copper grid and allowed to adhere for 10 s before being reduced to a thin film by blotting. Immediately after blotting, 3 μl of a 1% solution of uranyl formate was applied to the grid and blotted off directly. This was repeated three times. Data were acquired using a Tecnai Spirit transmission electron microscope operating at 120 kV. Images were processed using Relion 2.1^49^.

KcsA-BRIL and YiiP-BRIL complexes: Protein samples were diluted to 0.01–0.005 mg/mL and applied to freshly glow-discharged carbon-coated copper grids (400 mesh, EMS). Grids were then blotted, washed two times with 25 uL water droplets, and stained with two 25 μL droplets of 1% uranyl formate. Images were acquired at 49,000x magnification on a Tecnai F30 operating at 300 kV. At least 100 images were collected for each sample. KcsA-BRIL and YiiP-BRIL datasets, both with and without BAG2, were processed using EMAN2^50^ and Relion 3.0^51^. For the YiiP-BRIL-Bag2 dataset, the best 2D classes were selected and these particles were exported to cryoSPARC^52^ for model building. Three ab initio classes were generated and further subjected to heterogeneous refinement. The best class was then chosen for homogeneous refinement and crystal structures for BRIL–BAG2 (6CBV) and YiiP (3H90) were docked into the resulting map using Chimera^51^.

### CryoEM sample preparation and data collection

BRIL-3turn-KcsA/BAG2 complex: For cryoEM grid preparation C-Flat 1.2/1.3 400 mesh grids were glow discharged for 45 s at 25 mA. 3 µL of 4 mg/ml purified KcsA-BRIL–BAG2 complex in DDM was applied to each grid. Excess sample was removed by blotting in an FEI Vitrobot Mark IV at 100% RH and 4 °C using a Whatman #1 filter paper, a blot force of 2 and blot time of 7 s. Sample vitrification was accomplished by plunging into liquid nitrogen cooled liquid ethane. CryoEM data were collected at the University of Virginia Molecular Electron Microscopy Core (MEMC) on a Titan Krios equipped with a Thermo Fisher Scientific Falcon3EC detector operating with electron counting mode with a pixel size of 1.056 Å/pixel. 49 dose fractions were collected with 1.1 e^-^/Å^2^/fraction for a total dose of 54 e^-^/Å^2^. A total of 960 movies were collected.

α4(BRIL)β2-nAChR/BAK5 complex: 3.5 μL of 3 mg/ml α4(BRIL)β2-nAChR/BAK5 complex was applied to glow-discharged (0.39 mbar, −30 mA, 30 secs, in air) Quantifoil R1.2/1.3 200 mesh Au grids and vitrified using a Vitrobot Mark IV (ThermoFisher Scientific) at 100% RH and 4 °C. Frozen grids were loaded into a Titan Krios G2 transmission electron microscope (ThermoFisher Scientific) operating at 300 keV equipped with a Gatan K2 Summit direct electron detector. Images were recorded using SerialEM in super-resolution mode at a pixel size of 0.53 Å over a defocus range of −1.2 to 2.4 μm. Data were collected using a dose rate of 5.9 electrons per physical pixel per second, for 10.5 s total exposure, dose fractionated at 0.3 sec/frame (35 frames) corresponding to a total dose of 52 e^−^/Å^2^.

### Image processing and reconstruction of BRIL-3turn-KcsA/BAG2

Movies were motion corrected on the fly in Scipion v 1.2 with MotionCor2 v 1.0.2 with patch = 5 × 5 and with dose weighting^53,54^. The remainder of the processing was performed in RELION 3.0^51^. CTF estimations were calculated with Gctf v 1.18^55^. Image with Gctf maximum resolution estimates worse than 3.8 Å were excluded from further processing. Particles were picked using the RELION Laplacian-of-Gaussian model with minimum and maximum diameters of 100 and 180 Å. Particles were extracted with a box size of 220 pixels re-scaled to 110 pixels. Two rounds of 2D classification were performed to select good particles. A reference-free initial model was generated in RELION using C4 symmetry and used as the starting model for 3D classification with C4 symmetry and two classes.

### Image processing, model building, refinement of α4(BRIL)β2-nAChR/BAK5

Single-particle analysis was performed using the relion 3.0-beta workflow (Supplementary Figs. 6, 7)^51^. Movie frames were gain normalized, Fourier binned (to 1.102 Å/pixel), aligned, dose-weighted and summed using MotionCor2 v2.1^54^. Non-dose-weighted motion-corrected sums were used for estimation of defocus values using CTFFIND v4.0^56^. Particles (total of 1,867,100) were picked without a template using Gautomatch^57^, imported into relion and subjected to a round of 2D classification at 4.408 Å/pixel and box size = 80 pixels (Supplementary Fig. 6B). Classes showing distinct features (561,291 particles) were selected for a round of 3D classification at 2.204 Å/pixel using a low-pass filtered (60 Å) reference map, without symmetry imposed (C1) (Strategy 1). The reference map was generated from image processing of a small subset of the data using a low-pass filtered to 60 Å map of a published model of the α4β2 receptor (PDB: 6CNJ)^14^ as initial model. An initial model generated de novo from relion resulted in a similar map. All the resulting volumes from the first round of 3D classification show density for two sABs bound to the receptor, although one sAB appears to have weaker density. The particles corresponding to the volumes with the best resolution were selected for 3D autorefinement (total of 285,866) and then subjected to another round of 3D classification (round 2) without alignment. The resulting classes did not show differences in features with respect to the receptor, whereas variation was seen in the conformation of the sABs. The 3D classification without alignment was repeated with a mask focused on the receptor. The best map was selected and subjected to 3D autorefinement at 1.102 Å/pixel in relion to 4.2 Å. Bayesian polishing was performed, which improved the global resolution of the map to 3.9 Å. A round of CTF refinement further improved the map to 3.8 Å. Postprocessing (sharpening and masking focused on the receptor) in relion resulted in a map with overall resolution of 3.7 Å according to the FSC 0.143 criteria^58^.

Another round of image processing for the same dataset in relion 3.0-beta was performed to improve the BRIL-sAB region of the cryoEM map (Strategy 2, Supplementary Fig. 7). Particles (total of 285,902) from an earlier 3D autorefinement, prior to receptor-focused 3D classification, in the previous workflow were re-extracted with a bigger box size (200 pixel at 2.204 Å/pixel) and subjected to a round of 2D classification using a 350 Å diameter mask. The resulting class averages show that the diameter mask did not cut-off the sABs which still appear “blurred” suggesting high mobility, especially, of the constant regions of the sAB (Supplementary Fig. 6c). Good classes from the 2D classification were used for a focused 3D autorefinement without symmetry (1.102 Å/pixel, C1) using a generous mask including the receptor, micelle around the receptor, BRIL and sABs. Focused 3D classification (1.102 Å/pixel), without alignment, on each of the α4 subunits with their corresponding BRIL fusions and interacting sABs (regions masked are in red and blue) was performed in separate runs. When using masks focusing on the BRIL and the sAB only, the 3D classification did not result in a meaningful separation of classes. The best class from the each of the separate focused 3D classification was subjected to focused 3D autorefinement and postprocessing to yield maps with overall resolution of 3.9 Å and 4.0 Å, according to the FSC 0.143 criteria. The refined map prior to focused 3D classification on the α subunit-BRIL-sAB regions was also subjected to postprocessing to yield a map with overall resolution of 3.9 Å. Close inspection of the three final maps show very similar resolution (~4–5 Å) at the BRIL-sAB interface.

For the model refinement of the α4β2 receptor, the postprocessed map of the receptor only at 3.7 Å was used for real-space refinement in PHENIX^43^. The receptor chains, including the linked models for N-acetylglucosamine (NAG), from PDB ID: 6CNJ^14^ were placed into the cryoEM map by rigid-docking in Chimera^59^. Extra density was observed at the two known ligand binding sites in the receptor. The crystal structure of the 5HT binding protein (PDB ID: 5AIN) bound to varenicline^29^ was then superposed with the homologous extracellular domains of the α4β2 receptor (6CNJ) with an RMSD of 2.3 Å, thereby placing the varenicline in the said extra density. We then used the rigid-body docked merged models of the receptor and varenicline as initial model for real-space refinement in PHENIX. Several iterative rounds of building in COOT and real-space refinement in PHENIX were performed to refine the model of the varenicline-bound receptor. This refined model was then rigid-body docked to the postprocessed map of the receptor with visible density for BRIL and variable domains of sAB at 3.9 Å. Two copies of the crystal structure of BRIL–BAG2 (PDB ID: 6CBV) were also placed into the map by rigid-docking in Chimera. The three CDR-H1 residues different from BAG2 were modeled in COOT. Because of the poor density of the constant domains of the sABs, these regions in the model were removed prior to real-space refinement, but replaced by rigid-body docking prior to validation and deposition of the models. Data collection, refinement, and validation statistics are reported in Supp. Table 7. The coordinates and the maps are deposited in PDB with the codes 6UR8 and 6USF. Figures were prepared with UCSF Chimera and ChimeraX in SBGrid^59,60,61^.

### Statistics and reproducibility

Images from the micrographs related to Fig 2, Fig 5, Supplementary Fig 4 and Supplementary Fig 6 were collected until sufficient number of particles were imaged to perform the subsequent analyses.

### Reporting summary

Further information on research design is available in the Nature Research Reporting Summary linked to this article.

## Supplementary information

Supplementary Information

Reporting Summary

## Data Availability

The plasmids of the synthetic antibodies will be available from the corresponding author upon reasonable request. The crystal structure of the BRIL bound to the synthetic antibody BAG2 has the accession code 6CBV in Protein Data Bank. EM maps and atomic models of the cryoEM structure of the nicotinic acid receptor with the bound antibody BAK5 have been deposited at the Electron Microscopy data bank (Accession code: EMD-20863, EMD-20857) and the Protein Data Bank (Accession code: 6USF, 6UR8).
