# Supplementary material for: Synthetic antibodies against BRIL as universal fiducial marks for single−particle cryoEM structure determination of membrane proteins
Source: Nat Commun. 2020 Mar 27;11:1598. doi: 10.1038/s41467-020-15363-0 (PMC7101349; doi:10.1038/s41467-020-15363-0)
Supplement: Supplementary file 1 — Supplementary Information [file 41467_2020_15363_MOESM1_ESM.pdf]

# Synthetic antibodies against BRIL as “universal” fiducial marks for single-particle cryoEM structure determination of membrane proteins

Mukherjee et al

**Supplementary Figure 1**

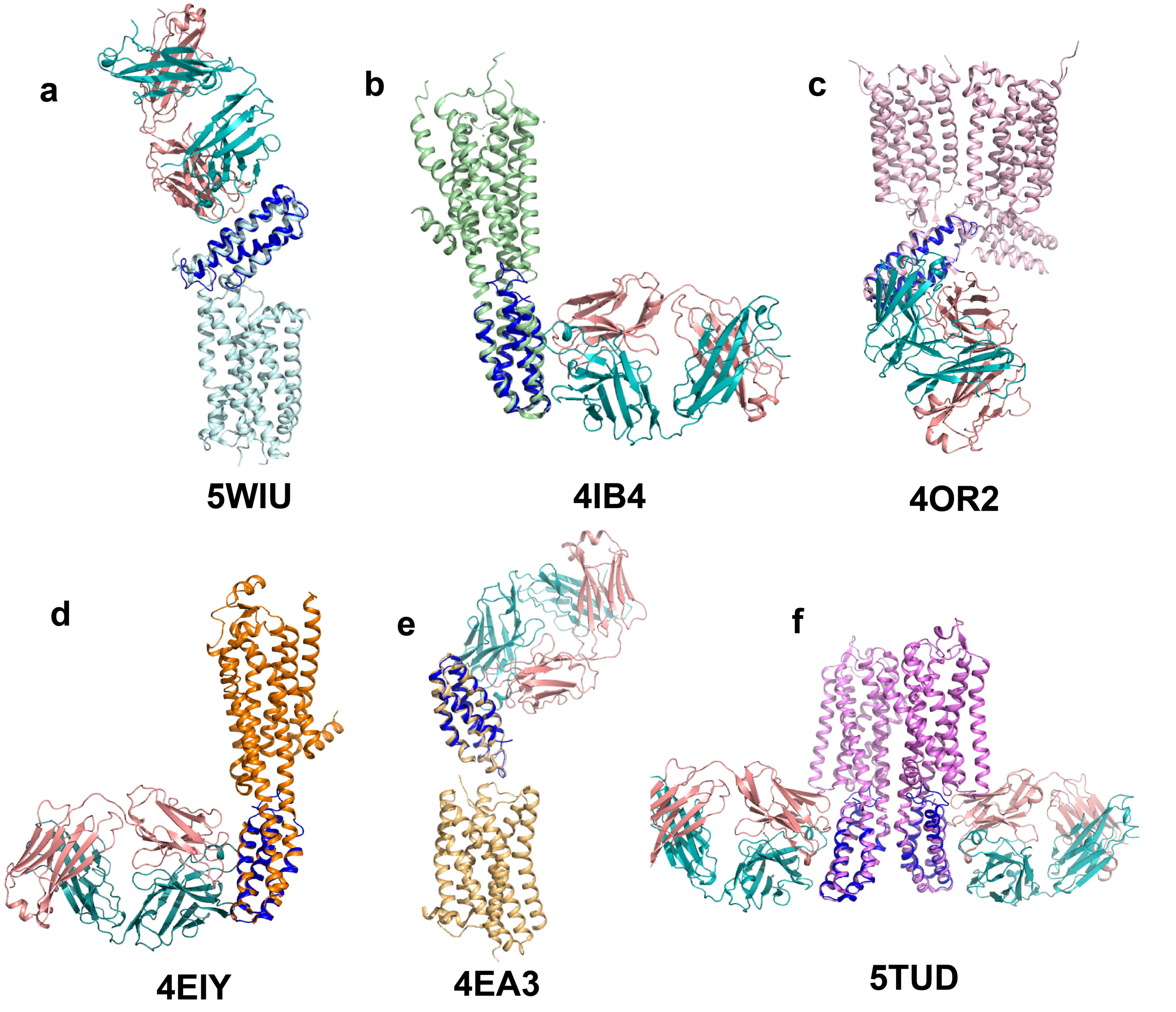


**Supplementary Figure 1: Compatibility of BAG2 in the BRIL-GPCR chimera:** Alignment of crystal structure of BRIL-BAG2 with crystal structures of different classes of GPCR with BRIL fusion either at the N-term or in the ICL3 position – **(a)** Dopamine D4 receptor (Class A) with N-term BRIL [PDB ID: 5WIU]. **(b)** Serotonin $5HT_{2B}$ receptor (Class A) with ICL3 BRIL [PDB ID: 4IB4] **(c)** Glutamate receptor I (Class C) with ICL3 BRIL [PDB ID: 4OR2]. **(d)** $A_{2A}$ adenosine receptor (Class A) with ICL3 BRIL [PDB ID: 4EIY]. **(e)** Opioid N/OFQ receptor (Class A) with N-term BRIL [PDB ID: 4EA3]. **(f)** Serotonin $5HT_{2B}$ receptor (Class A) with ICL3 BRIL [PDB ID: 5TUD]. In all cases, no potential clash was observed between the receptor and BAG2-BRIL structure.

**Supplementary Figure 2**

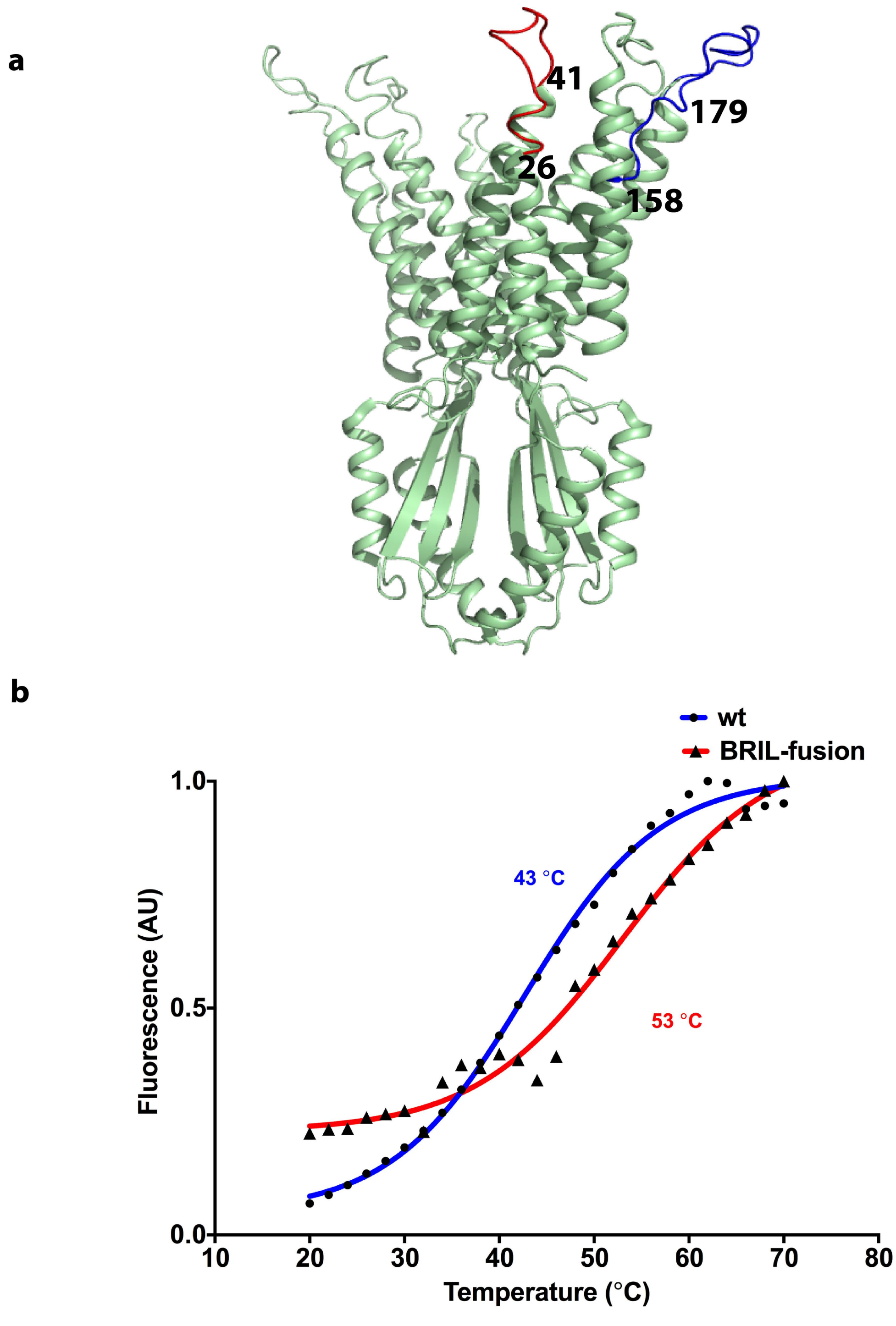


**Supplementary Figure 2: Design and characterization of the YiiP-BRIL fusion constructs: (a)** Two different loops – loop (residues 26-41, colored red) connecting helices 1 and 2 and loop (residues 158-179, colored blue) connecting helices 5 and 6 were tested for BRIL insertion. **(b)** Overlaid thermal melting curves of wt YiiP and the YiiP-BRIL fusion monitored by fluorescence using CPM probe.

**Supplementary Figure 3**

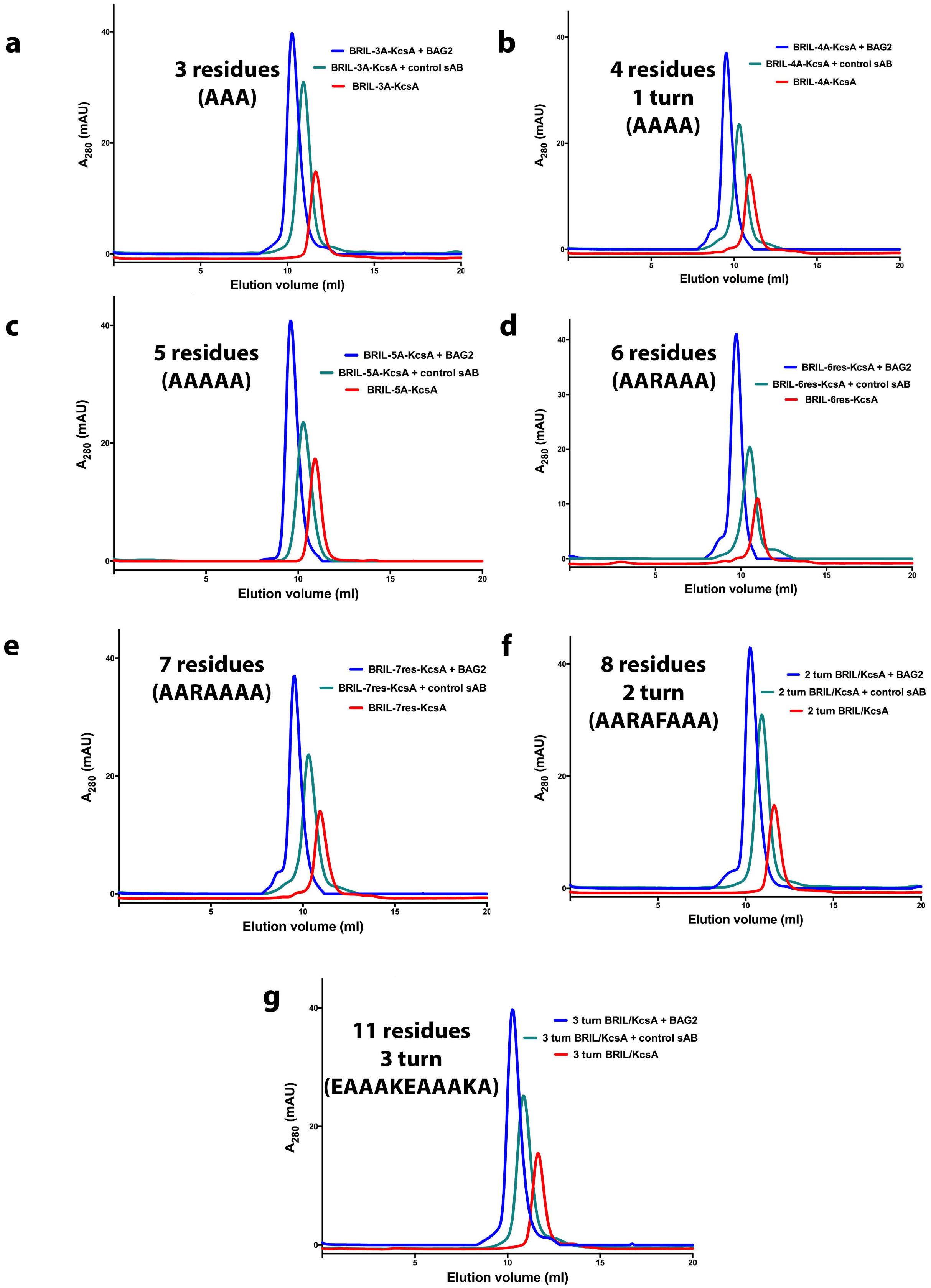


**Supplementary Figure 3: BAG2 binding to different KcsA-BRIL fusion constructs:** aSEC curves of the BRIL-KscA fusion constructs with different helical linkers without any sAB (red), with BAG2 (blue) and with a control sAB (green). In all constructs, 4 copies of BAG2 bind to the tetrameric assembly of KcsA as evident from the shift in the elution peak in comparison to that of the complex with control sAB. 2 copies of control sAB bind to each tetramer of KcsA (unpublished data).

**Supplementary Figure 4**

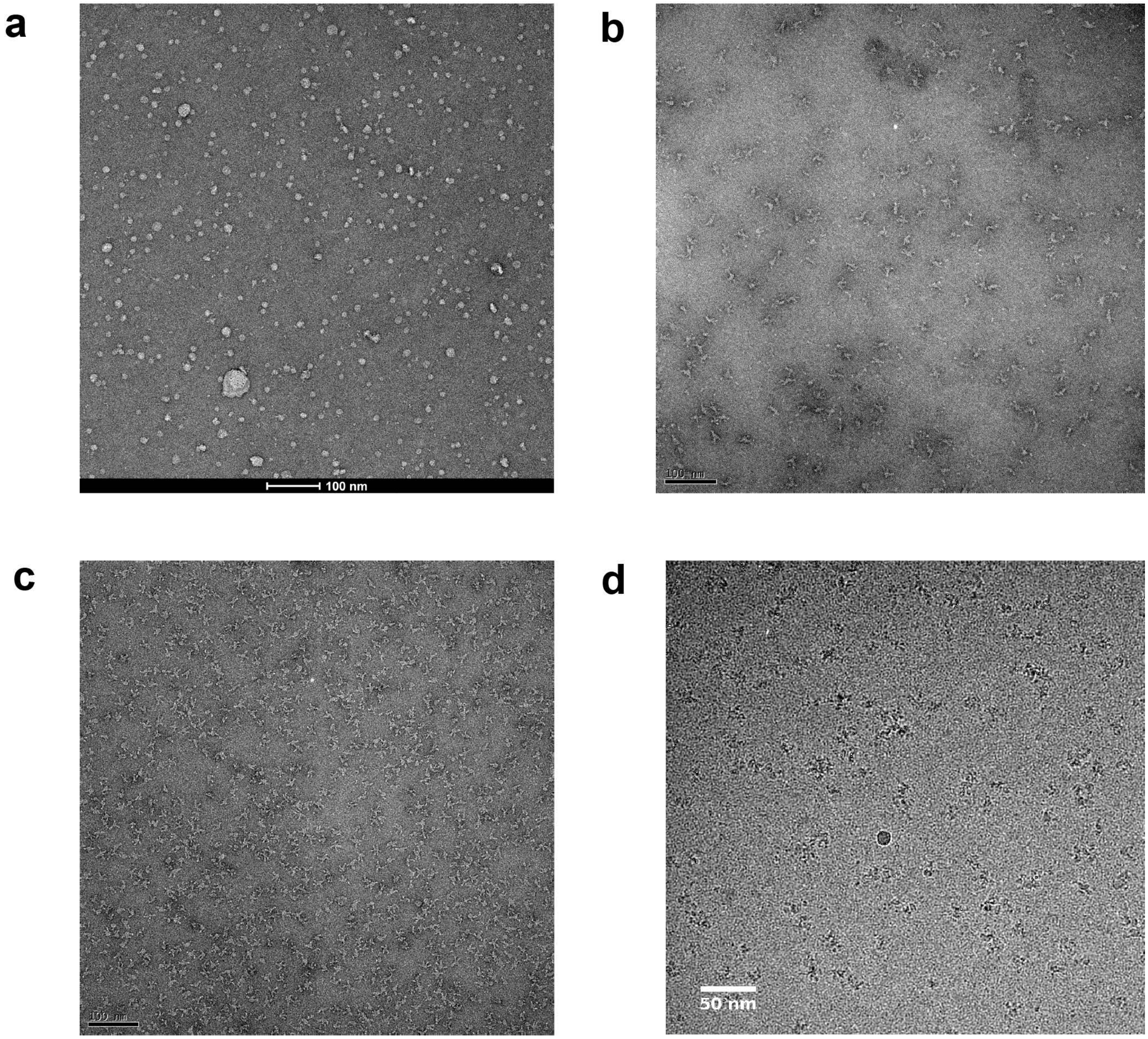


**Supplementary Figure 4: Representative micrographs of sAB bound membrane protein targets:** Negative stain micrographs of complex of **(a)** Serotonin $5HT_{1B}$ receptor and sAB24. **(b)** YiiP-BRIL and BAG2. **(c)** BRIL-2turn-KcsA and BAG2. **(d)** CryoEM micrograph of BRIL-3turn-KcsA bound to BAG2.

**Supplementary Figure 5**

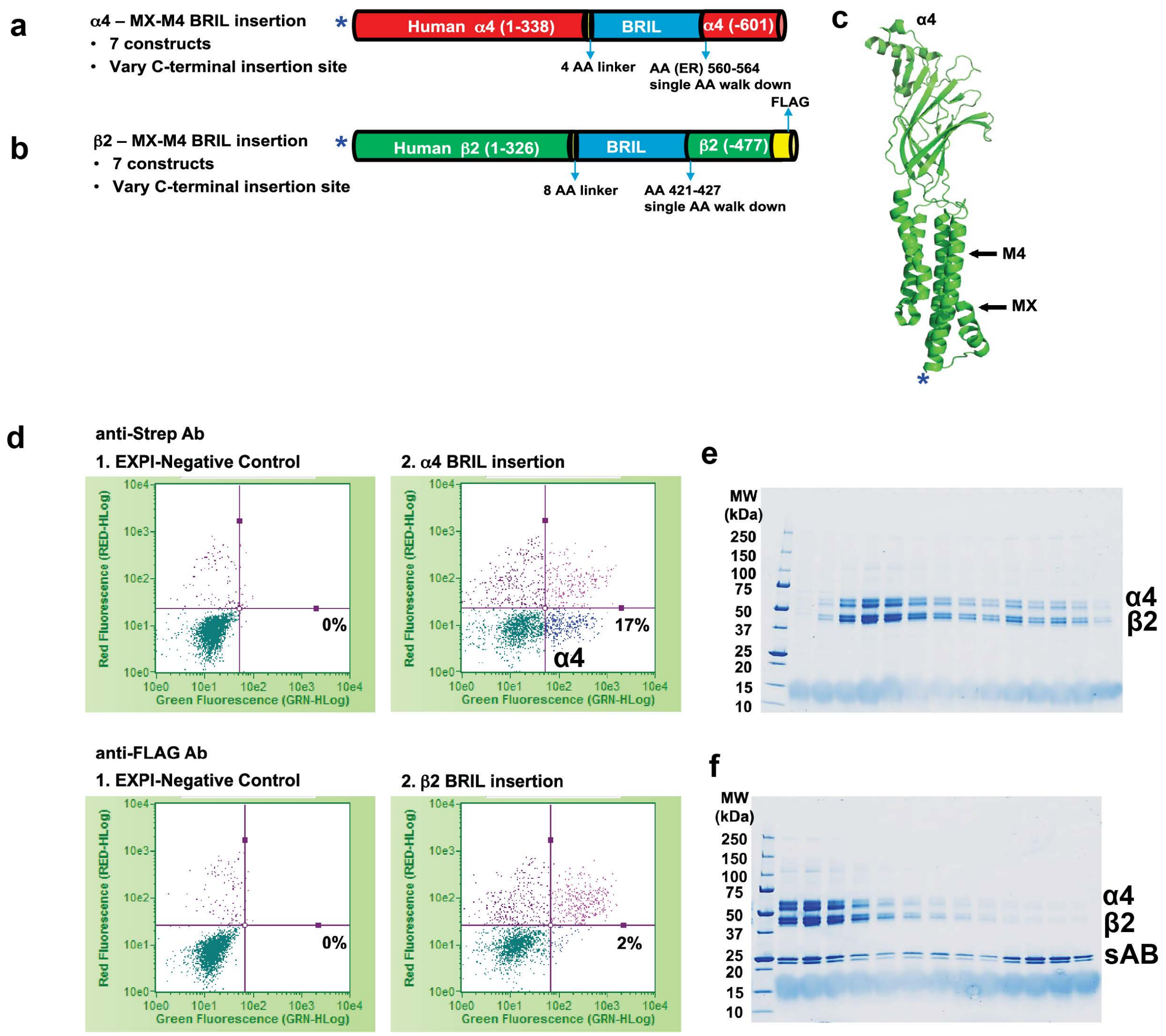


**Supplementary Figure 5: Design and characterization of α4β2 nAChR BRIL fusion constructs: (a)** BRIL was inserted between the MX and M4 helices α4, with 4 residues added before the N-terminus of BRIL as a linker. Seven constructs of α4-fusion were made by varying the fusion site after the C-terminus of BRIL by 7 single residue deletions (-ER-560-564) into the C-terminus of α4. **(b)** BRIL was inserted between the MX and M4 helices in β2 adding, 8 residues before the N-terminus of BRIL as a linker. Seven constructs of β2 were made by varying the fusion site after the C-terminus of BRIL by 7 single residue deletions (421-427) into the C-terminus of β2. **(c)** The BRIL fusion location in α4 between MX and M4 helices is marked by an asterisk. **(d)** Guava assay of 2 constructs representing α4 and β2 BRIL fusions. Expression of the receptor at the cell surface was detected using antibodies to the affinity tags on the subunits (top panel probed with anti-Strep antibody, bottom panel probed with anti-FLAG antibody). The lower right quadrant in each plot is indicative of protein expression at the cell surface. BRIL insertion in α4 has higher expression (17%) compared to that in β2 (2%). **(e)** SDS-PAGE analysis of the fractions obtained from streptactin affinity chromatography purification of construct 8 with BRIL fusion α4 subunit, shows the β2 subunit band is denser, indicating that it is present in molar excess to α4 subunit. This suggests that subunit stoichiometry is 2:3 (α4:β2). **(f)** SDS-PAGE analysis of the SEC fractions from the construct 8-sAB complex shows co-elution of the sAB with the receptor confirming the complex formation. The excess sAB used in the complex formation elutes later from the column.

**Supplementary Figure 6**

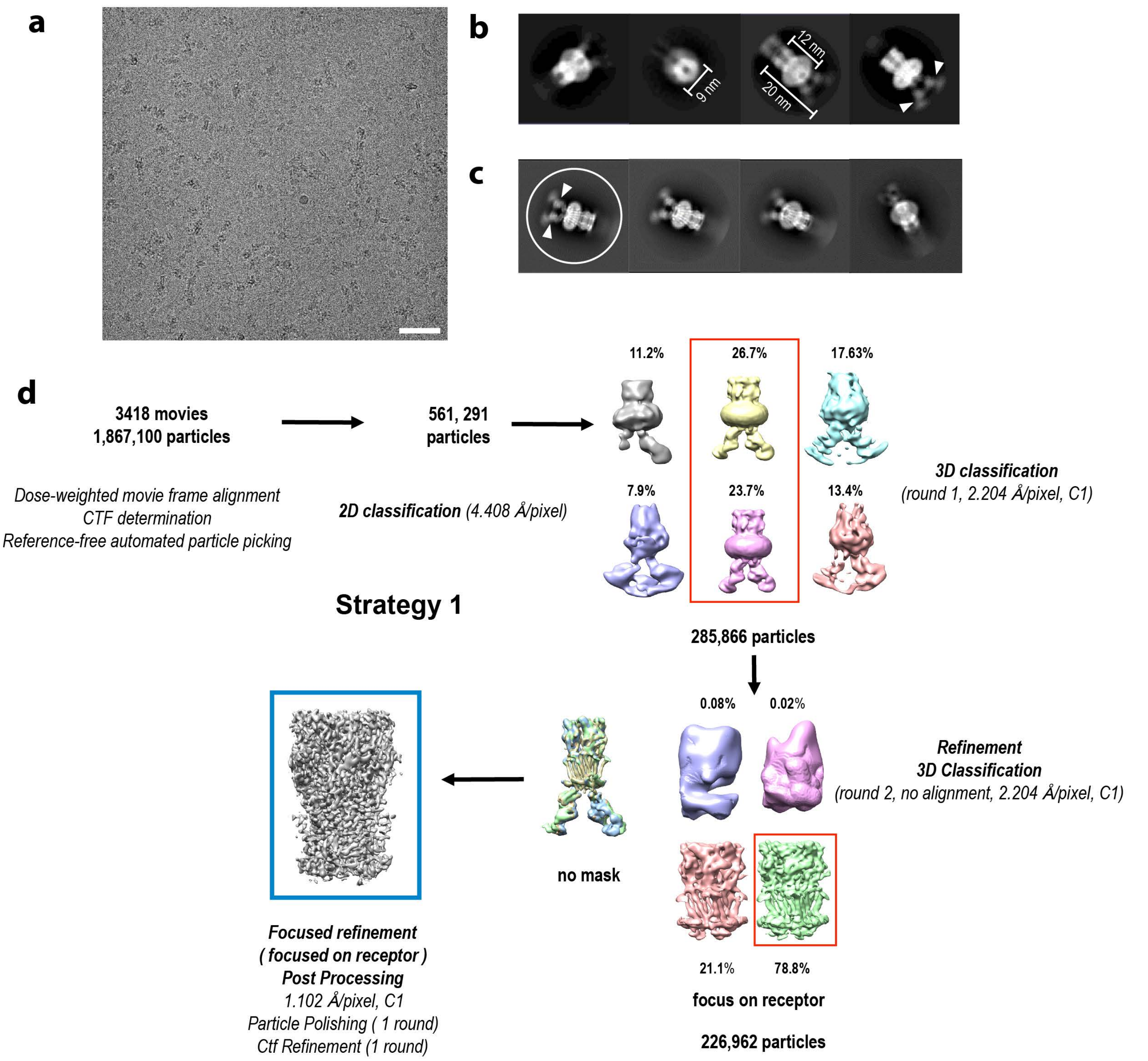


**Supplementary Figure 6: CryoEM data, 2D classification analyses and workflow of BRIL fused α4β2 nAChR bound to BAK5: (a)** Representative aligned sum of a movie of the receptor-sAB complex. Scale bar, 50 nm. **(b)** Selected 2D class averages from 1st round of 2D classification. The dimensions of the receptor and the receptor-sAB complex are shown. Arrows indicate the location of the sABs. **(c)** Selected 2D class averages of re-centered and re-extracted particles to refine the density of the BRIL andsAB regions. Arrows indicate the location of the sAB. A white circle outlines the diameter (350 Å) used for masking during 2D classification. **(d)** Strategy 1 - CryoEM data processing workflow focusing on the refinement of the α4β2 receptor only. Data processing procedure was implemented in relion-3.0-beta. The final map (blue box) has been sharpened and masked focusing on the α4β2 receptor.

**Supplementary Figure 7**

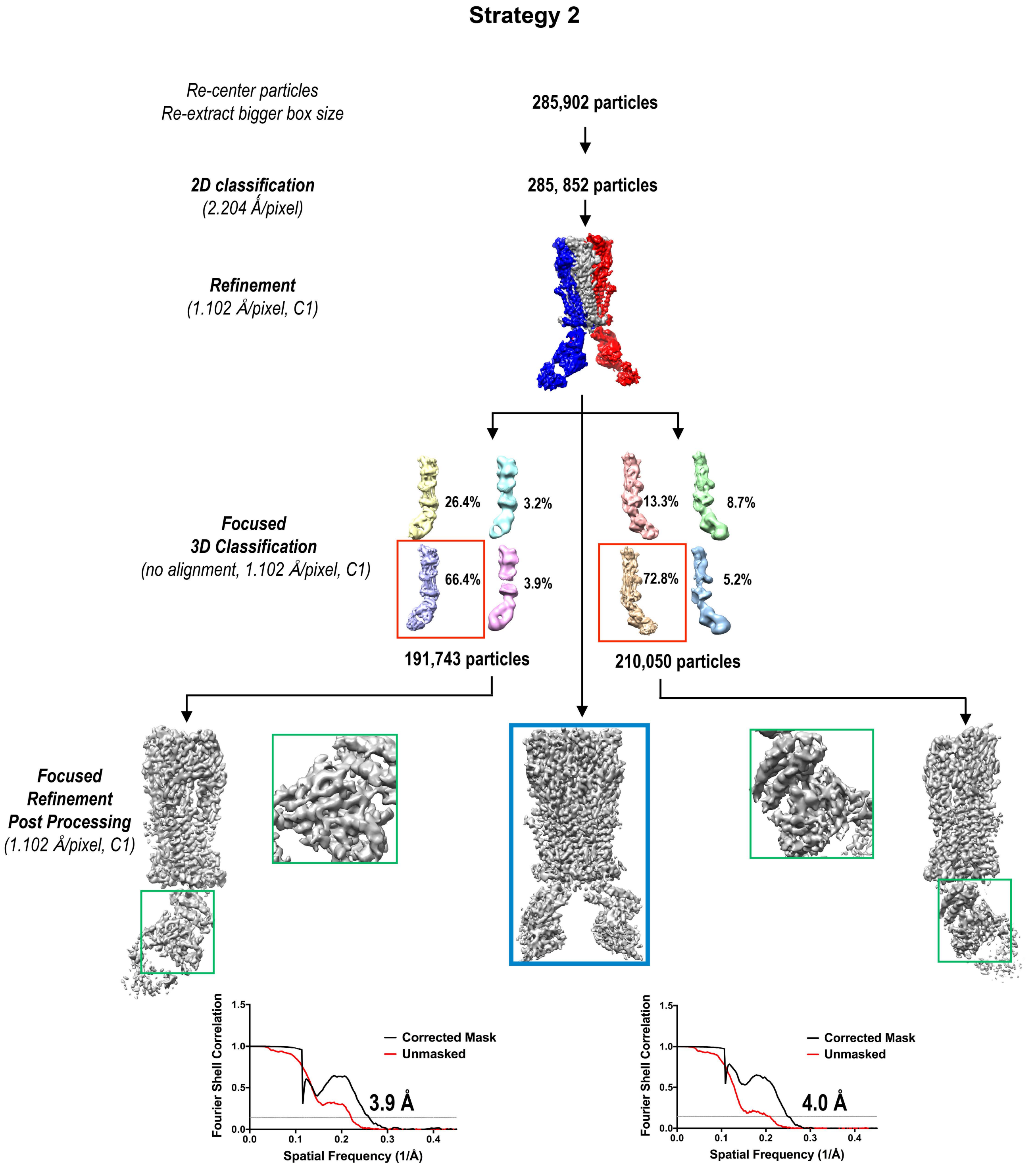


**Supplementary Figure 7: CryoEM data processing workflow for improving the BRIL-BAK5 region of the α4β2-BAG2 complex.** Strategy 2 - Data processing procedure was implemented in relion-3.0-beta. The refined map (final) skipping focused 3D classification (blue box) has been sharpened and masked. Focused 3D classification was performed with mask on either α subunits including the BRIL and the sAB. The resulting post-processed maps with accompanying FSC curves are also shown. Close-up view of the BRIL-BAK5 interface is shown (green box).

**Supplementary Figure 8**

## Results from Strategy 1 (from Suppl. Fig. 6d)

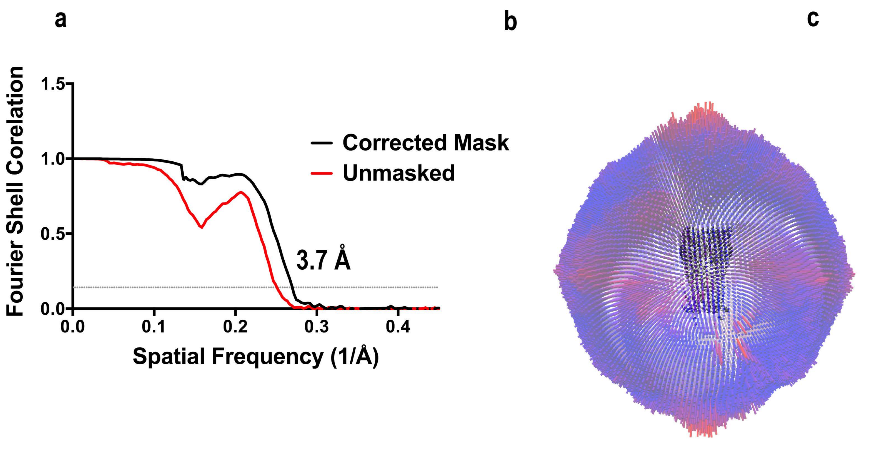


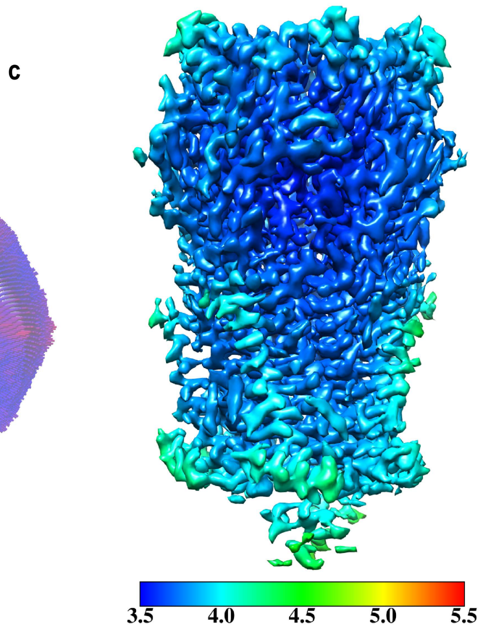


## Results from Strategy 2 (from Suppl. Fig. 7)

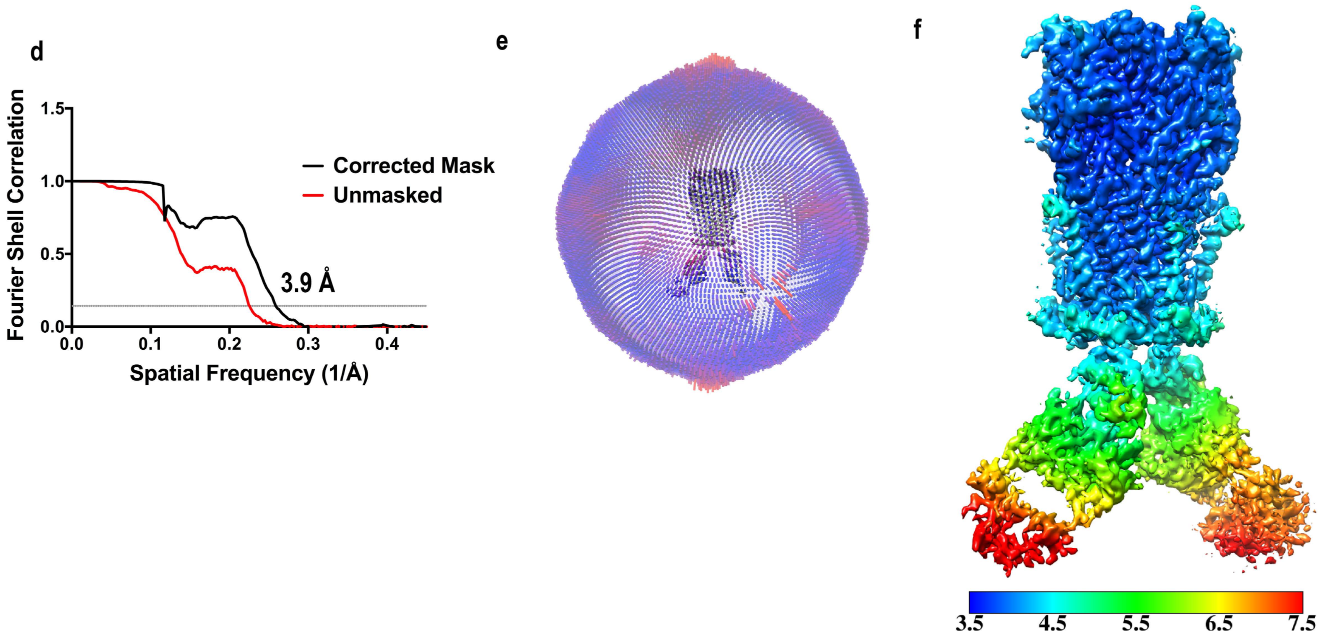


**Supplementary Figure 8: FSC Curves, angular distribution histogram and local resolution maps:** FSC curves of the unmasked (red) and corrected masked (black) map with the indicated global resolution **(a)** at 3.7 Å (dotted) using Strategy 1 and **(d)** at 3.9 Å (dotted) using Strategy 2. Angular distribution histogram of the **(b)** refined map using Strategy 1 and **(e)** the final map using Strategy 2. Local resolution map **(c)** using Strategy 1 and **(f)** using Strategy 2 with the color keys in Å. The workflow using Strategy 1 and 2 have been detailed in Suppl. Fig. 6d and Suppl. Fig. 7 respectively.

**Supplementary Figure 9**

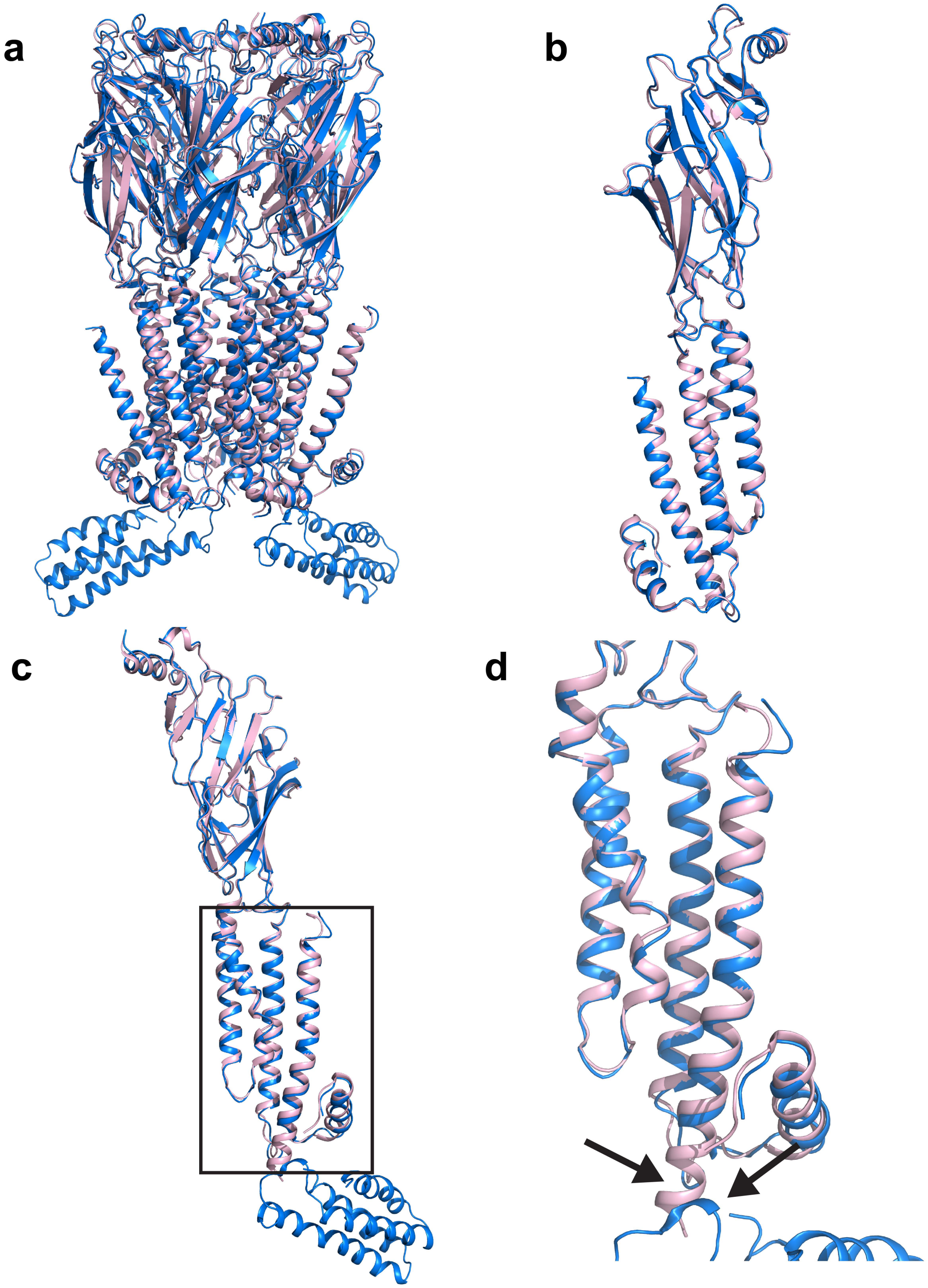


**Supplementary Figure 9:** Superposition of α4β2 nAChR with and without BRIL fusion: **(a)** Cryo-EM α4β2 nAChR without BRIL fusion (PDB ID: 6CNJ, colored light pink) was superposed with the cryo-EM structure of the α4β2 nAChR with BRIL fusion under study (PDB ID: 6USF, colored marine) with an RMSD $C_\alpha$ = 0.7Å. **(b)** Superposition of the β2 subunit. **(c)** Superposition of the α4 subunit. **(d)** Close-up view of the boxed part of (c). RMSD $C_\alpha$ upon superposition of the helices (6USF) into which BRIL was fused with that of 6CNJ was 0.3 Å (residues 278-306, numbering based on 6CNJ) and 0.5 Å (residues 355-370, numbering based on 6CNJ). The points of attachment of BRIL in between the helices are shown by black arrows.

**Supplementary Table 1: CDR sequences of sABs and kinetic parameters of binding to BRIL by SPR**

| sAB ID | $k_{on}$ ($M^{-1}s^{-1}$) | $k_{off}$ ($s^{-1}$) | $K_D$ (nM) |
|---|---|---|---|
| sAB1 | $6.0 \times 10^{4}$ | $3.6 \times 10^{-4}$ | 6.0 |
| sAB2 | $5.1 \times 10^{4}$ | $9.4 \times 10^{-5}$ | 1.9 |
| sAB7 | $4.7 \times 10^{5}$ | $5.7 \times 10^{-3}$ | 12.2 |
| sAB10 | $1.6 \times 10^{5}$ | $2.0 \times 10^{-3}$ | 12.5 |
| sAB12 | $1.6 \times 10^{5}$ | $1.6 \times 10^{-4}$ | 1.0 |
| sAB18 | $1.3 \times 10^{5}$ | $1.9 \times 10^{-3}$ | 14.0 |
| sAB24 | $3.4 \times 10^{4}$ | $8.0 \times 10^{-4}$ | 23.0 |

**Supplementary Table 2: Kinetic parameters of the wild type (wt) and affinity matured variants of sAB24 determined by SPR**

| sAB ID | CDR-L3 | CDR-H1 | CDR-H2 | CDR-H3 | $k_{on}$ ($M^{-1}s^{-1}$) | $k_{off}$ ($s^{-1}$) | $K_D$ |
|---|---|---|---|---|---|---|---|
| **wt sAB24** | YLYYSLV | FSSSSI | YISSSSGSTS | WGYWPGEPWWKAF | $3.4 \times 10^{4}$ | $8.0 \times 10^{-4}$ | 23.0 nM |
| **BAG2** | | VVDFSL | | | $4.9 \times 10^{5}$ | $1.3 \times 10^{-4}$ | 269 pM |
| **BAK7** | | VIGFTI | | | $4.2 \times 10^{5}$ | $1.9 \times 10^{-4}$ | 446 pM |
| **BAK5** | | VRSFSI | | | $7.8 \times 10^{5}$ | $1.3 \times 10^{-4}$ | 175 pM |
| **BAG5** | | ISEYTV | | | $6.3 \times 10^{5}$ | $9.8 \times 10^{-5}$ | 155 pM |

CDR-L3, H2 and H3 sequences of the wt sAB24 and the affinity matured variants are identical.

## Supplementary Table 3: Kinetic parameters of Alanine scanning mutants

| Fab ID | CDR-H1 | $k_{on}$ ($M^{-1}s^{-1}$) | $k_{off}$ ($s^{-1}$) | $K_D$ (nM) |
|---|---|---|---|---|
| wt sAB-24 | FSSSSI | $3.4 \times 10^{4}$ | $8.0 \times 10^{-4}$ | 23.0 |
| BAG2 | VVDFSL | $4.9 \times 10^{5}$ | $1.3 \times 10^{-4}$ | 0.3 |
| F35A | VVDASL | $3.8 \times 10^{4}$ | $2.8 \times 10^{-4}$ | 7.6 |
| S36A | VVDFAL | $7.0 \times 10^{4}$ | $3.7 \times 10^{-4}$ | 5.3 |

## Supplementary Table 4: Data collection and refinement statistics

Statistics for the highest-resolution shell are shown in parentheses.

| | |
|---|---|
| **Data Collection** | |
| Space Group | $P6_5$ |
| Cell dimensions | |
| a, b, c (Å) | 87.95, 87.95, 159.11 |
| α, β, γ (°) | 90, 90, 120 |
| Resolution (Å) | 20.0 - 1.87 (1.99 - 1.87) |
| Rmerge (%) | 4.1 (89.2) |
| $CC_{1/2}$ | 0.99 (0.46) |
| $\langle I \rangle / \langle \sigma(I) \rangle$ | 17.55 (0.94) |
| Completeness (%) | 96.7 (81.6) |
| Redundancy | 4.8 (2.5) |
| **Refinement** | |
| Resolution (Å) | 20.0 - 1.87 (1.99 - 1.87) |
| Reflections | 55218 (4117) |
| $R_{cryst}$ (%) | 19.4 |
| $R_{free}$ (%) | 22.5 |
| Number of atoms | |
| Protein | 4169 |
| Ligands/Ions | 19 |
| Water | 320 |
| Average B-factor (Å$^2$) | |
| Protein | 56.2 |
| Ligands/Ions | 52.4 |
| Water | 53.0 |
| RMSD | |
| Bond Lengths (Å) | 0.005 |
| Bond Angles (°) | 1.1 |
| Ramachandran Plot Statistics | |
| Favored (%) | 97.8 |
| Allowed (%) | 2.2 |
| Outliers (%) | 0.0 |

**SupplementaryTable 5: Hydrogen bonds and salt bridges between BRIL and BAG2**

| BRIL | BAG2 | Distance (Å) |
| --- | --- | --- |
| | **Light Chain** | |
| Arg34[NH1] | Leu93[O] | 2.9 |
| Asp39 [N] | Ser31[OG] | 3.5 |
| Asp39[OD1] | Arg67[NH1] | 2.8 |
| Asp39[OD1] | Ser31[OG] | 2.5 |
| Asp39[OD2] | Arg67[NH2] | 2.7 |
| Asp39[OD1] | Arg67[NH2] | 3.5 |
| Asp39[OD1] | Arg67[NH1] | 2.8 |
| Asp39[OD2] | Arg67[NH2] | 2.7 |
| Asp39[OD2] | Arg67[NH1] | 3.6 |
| Asn80[ND2] | Ser96[OG] | 3.0 |
| | **Heavy Chain** | |
| Asp66[O] | Trp105[NE1] | 3.0 |
| Asn80[ND2] | Ser62[OG] | 3.9 |

**SupplementaryTable 6: (a) Design and (b) characterization of the different b-RIL-fused constructs of human α4β2 nAChR**

### Supplementary Table 6(a)

| Subunits | Construct ID of individual subunits | BRIL fusion | C-term of BRIL fused at[1] |
|---|---|---|---|
| α4 | A | None | NA |
| | B | N-term of α4 | 1 |
| | C | α4 insertion | -ER-560 |
| | D | α4 insertion | -R-560 |
| | E | α4 insertion | -560 |
| | F | α4 insertion | -561 |
| | G | α4 insertion | -562 |
| | H | α4 insertion | -563 |
| | I | α4 insertion | -564 |
| β2 | J | None | NA |
| | K | β2 insertion | -421 |
| | L | β2 insertion | -422 |
| | M | β2 insertion | -423 |
| | N | β2 insertion | -424 |
| | O | β2 insertion | -425 |
| | P | β2 insertion | -426 |
| | Q | β2 insertion | -427 |

[1]The C-terminus of BRIL was fused to the subunit residue listed.

## SupplementaryTable 6(b)

| Final construct pair ID | Pair from construct ID of individual subunits[1] | BRIL fusion at | Small Scale expression[2] (%) | Large Scale expression[2] (%) | Monodispersity on aSEC |
|---|---|---|---|---|---|
| 1 | A + J | None | 35.6 | NA | NA |
| 2 | B + J | N-term of α4 | 22.9 | 20 | Aggregation |
| 3 | C + J | α4 insertion | 16.3 | NA | NA |
| 4 | D + J | | 18.2 | NA | NA |
| 5 | E + J | | 15.9 | NA | NA |
| 6 | F + J | | 16.8 | NA | NA |
| 7 | G + J | | 18.8 | NA | NA |
| 8 | H + J | | 19.7 | 17 | Monodisperse |
| 9 | I + J | | 16.5 | NA | NA |
| 10 | A + K | β2 insertion | 20.7 | NA | NA |
| 11 | A + L | | 19.2 | NA | NA |
| 12 | A + M | | 16.3 | NA | NA |
| 13 | A + N | | 24.5 | 2 | Monodisperse |
| 14 | A + O | | 18.8 | NA | NA |
| 15 | A + P | | 18.5 | NA | NA |
| 16 | A + Q | | 13.1 | NA | NA |

[1] Each α4 BRIL fusion construct (pairs 2-9) was paired with the β2 subunit [construct ID: J from previous table 6(a)] without any BRIL fusion. Similarly each β2 BRIL fusion construct (pairs 10-16) was paired with the α4 subunit [construct ID: A from previous table 6(a)] without any BRIL fusion. [2] Expression was measured in the Guava assay in the presence of nicotine.

## Supplementary Table 7: Cryo-EM data collection, refinement, and validation statistics

| **Data Collection and Processing** | **Receptor only map** | **Full map with BAG2** |
|---|---|---|
| Microscope | FEI Titan Krios G2 | FEI Titan Krios G2 |
| Magnification | ×22,500 | ×22,500 |
| Voltage (kV) | 300 | 300 |
| Electron exposure ($e^{-}$ Å$^{-2}$) | 52 | 52 |
| Defocus range (µm) | -1.5 – -2.5 | -1.5 – -2.5 |
| Pixel size (Å) super-resolution mode | 0.5512 | 0.5512 |
| Symmetry imposed | C1 | C1 |
| Initial particle images | 1,867,100 | 1,867,100 |
| No. of particles in final map | 226962 | 285852 |
| Map resolution (Å) | 3.7 | 3.9 |
| FSC threshold | 0.143 | 0.143 |
| Map resolution range (Å) | 3.6 – 4.6 | 3.6 – 8.6 |
| **Refinement** | | |
| Initial model used (PDB code) | 6CNJ, 5AIN | 6CNJ, 6CBV |
| Model resolution cut-off (Å) | 3.8 | 4.0 |
| FSC threshold | 0.5 | 0.5 |
| Map sharpening *B* factor (Å$^2$) | –120 | –162 |
| Model Composition | | |
| Non-hydrogen atoms | 14,880 | 23022 |
| Protein residues | 1798 | 2868 |
| Ligands | 13 | 13 |
| B-factors (Å$^2$) | | |
| Protein | 19.8 | 77.6 |
| Ligands | 45.6 | 96.7 |
| RMSD | | |
| Bond lengths (Å) | 0.01 | 0.02 |
| Bond angles (°) | 1.3 | 1.7 |
| **Validation** | | |
| MolProbity score | 1.7 | 2.0 |
| Clashscore | 4.2 | 10.4 |
| Poor rotamers (%) | 0.0 | 0.08 |
| Ramachandran plot | | |
| Favored (%) | 93.08 | 93.04 |
| Allowed (%) | 6.92 | 6.96 |
| Outliers (%) | 0.0 | 0.0 |
| PDB ID | 6UR8 | 6USF |
